# Traditional chinese medicine in coronary microvascular disease

**DOI:** 10.3389/fphar.2022.929159

**Published:** 2022-08-08

**Authors:** Zhihua Yang, Shanshan Lin, Yangxi Liu, Qiuan Ren, Zhao Ge, Ci Wang, Yingfei Bi, Xianliang Wang, Jingyuan Mao

**Affiliations:** ^1^ National Clinical Research Center for Chinese Medicine Acupuncture and Moxibustion, First Teaching Hospital of Tianjin University of Traditional Chinese Medicine, Tianjin, China; ^2^ Institute of Traditional Chinese Medicine, Tianjin University of Traditional Chinese Medicine, Tianjin, China

**Keywords:** traditional Chinese medicine, clinical evidence, coronary microvascular disease, coronary microvascular dysfunction, coronary flow reserve, potential mechanism

## Abstract

Coronary microvascular disease (CMVD) is common in patients with cardiovascular risk factors and is associated with an increased risk of adverse cardiovascular events. Although the study of CMVD in modern medicine is ongoing, there is still no effective treatment for it. Traditional Chinese medicine (TCM) has some clinical advantages based on syndrome differentiation and individualized treatment. In this review, we review the clinical significance, pathogenesis, and current treatments of CMVD and systematically summarize the clinical efficacy and potential action mechanisms of TCM for CMVD. In addition, the scientific problems that need to be solved urgently and the research strategy of TCM for CMVD are described. CMVD has great clinical significance, but there are still many gaps in the related research. This review aims to attract the attention of clinicians to CMVD and promote research on CMVD in TCM.

## 1 Introduction

**FIGURE 1:**
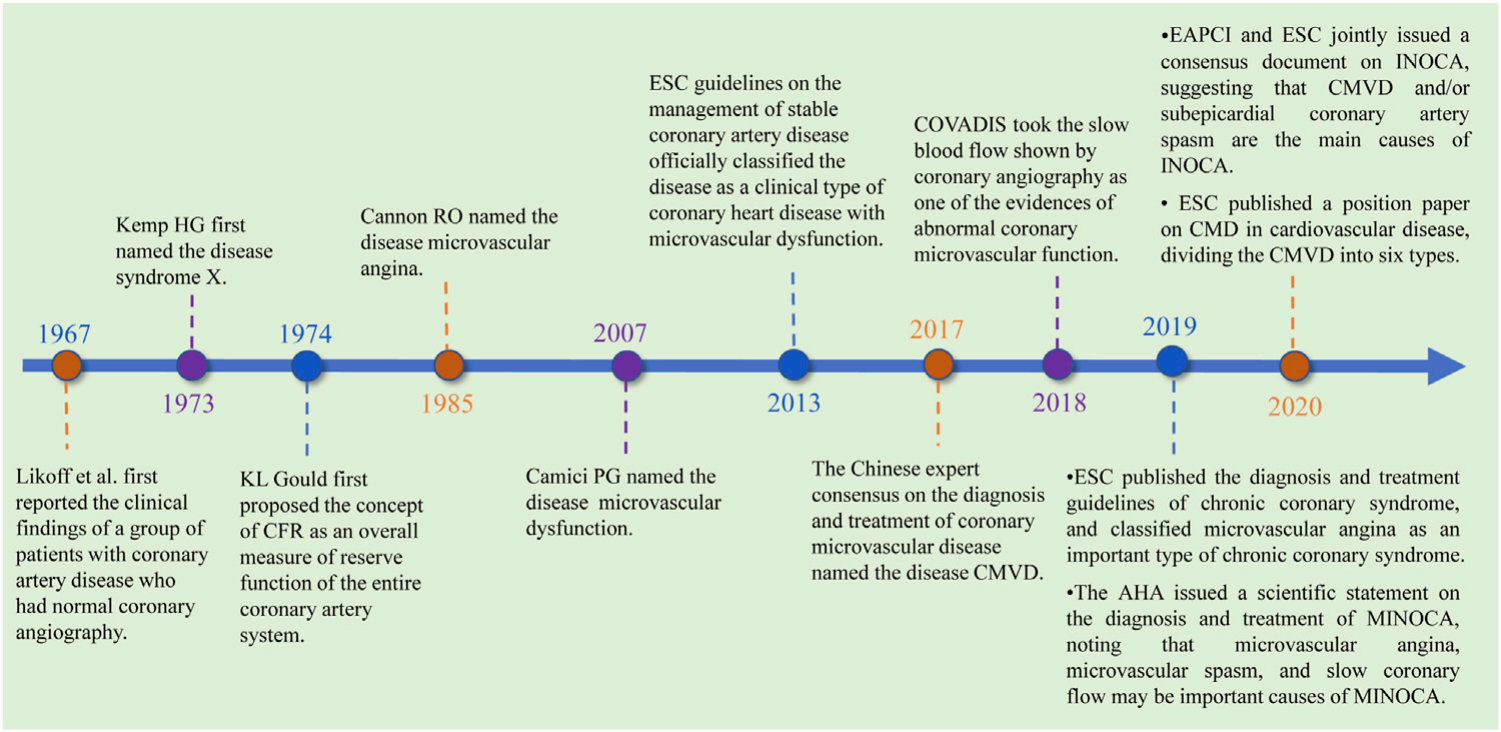
Development and research progress of CMVD.

ESC, European Society of Cardiology; COVADIS, Coronary Vasomotion Disorders International Study Group; MINOCA, Myocardial Infarction with No-Obstructive Coronary Arteries; EAPCI, European Association of Percutaneous Cardiovascular Interventions; INOCA, Ischemia and No Obstructive Coronary Artery Disease.

Coronary microvascular disease (CMVD) is a clinical syndrome that exhibits objective evidence of exertional angina pectoris or myocardial ischemia caused by structural and/or functional abnormalities of the coronary artery microvasculature under the action of many pathogenic factors (Zhang et al., 2017). The disease tends to occur in women (Crea et al., 2014; Murthy et al., 2014; Sara et al., 2015; Mileva et al., 2022) and is also prevalent in certain populations, such as those with obesity, hypertension, insulin resistance, diabetes, metabolic syndrome, smoking, myocardial injury, cardiomyopathy, ejection fraction-preserved heart failure, and obstructive and nonobstructive coronary heart disease (Crea et al., 2014; Labazi and Trask, 2017; Taqueti and Di Carli, 2018). In addition, the incidences of major adverse cardiovascular events (MACEs) and all-cause mortality associated with the disease are high (Murthy et al., 2014). In 1973, Kemp HG first referred to CMVD as syndrome X (Kemp et al., 1973). In 1985, Cannon RO referred to the disease as microvascular angina (Cannon et al., 1985). In 2007, Camici PG referred to the disease as microvascular dysfunction. In 2013, the European Society of Cardiology (ESC) issued guidelines that formally named the disease coronary microvascular dysfunction and defined it as one of the types of stable coronary heart disease (Task Force et al., 2013). In 2017, the *Chinese Expert Consensus on Diagnosis and Treatment of Coronary Microvascular Disease* called the disease “coronary microvascular disease” (Zhang et al., 2017), and CMVD was divided into three types: 1) coronary artery disease without obstruction; 2) coronary artery disease with obstruction; and 3) other types of CMVD, including hypertrophic cardiomyopathy, dilated cardiomyopathy, myocarditis, aortic stenosis, and myocardial amyloidosis. In 2020, the ESC Working Group on Coronary Pathophysiology and Microcirculation published a paper on “coronary microvascular dysfunction in cardiovascular disease,” in which CMVD was divided into 1) CMVD in nonobstructive chronic coronary syndromes, 2) CMVD in obstructive chronic coronary syndromes, 3) CMVD in nonobstructive acute coronary syndromes (ACS), 4) CMVD in obstructive ACS, and 5) CMVD and coronary no-reflow CMVD in reperfused acute myocardial infarction (AMI). The last of these has recently been recognized as the major cause of angina or heart failure following successful reperfusion therapy in AMIs (Padro et al., 2020). In recent years, the study of CMVD has been expanded, and its development and progress are shown in Figure 1.

Coronary artery disease is the leading cause of death worldwide (Khan et al., 2020). Recently, the development of thrombolysis and percutaneous coronary interventions (PCI) has solved the problem of coronary artery stenosis or occlusion to a great extent. It was found that even when adequate and timely reperfusion therapy restored epicardial coronary blood flow, CMVD still occurred in many patients (Niccoli et al., 2016). At present, the main therapeutic drugs used for CMVD are mainly anti-myocardial ischemia drugs, which include beta-blockers, short-acting nitrates, angiotensin-converting enzyme inhibitors (ACEI), angiotensin receptor blockers (ARB), calcium channel blockers (CCB), ivabradine, trimetazidine, and ranolazine (Task Force et al., 2013; Villano et al., 2013; Mumma and Flacke, 2015; Bairey Merz et al., 2016; Shah et al., 2017). Although these drugs have certain clinical efficacy, many patients still require repeated hospitalization and coronary angiography because of chest pain, which seriously affects patients’ quality of life (Zhuang et al., 2020). Therefore, more effective therapies for CMVD are still needed (Marinescu et al., 2015; Vink et al., 2021). Clinical studies have shown that TCM has good clinical efficacy in treating CMVD and can significantly improve the symptoms of angina pectoris, TCM syndrome, ECG ischemia, quality of life, exercise tolerance, and other related physical and chemical indicators (Zhong et al., 2020; Wang M. X. et al., 2021). In this study, the clinical significance, pathogenesis, and current treatments of CMVD, as well as the clinical efficacy and mechanism of TCM for CMVD, are systematically summarized and discussed to attract the attention of clinicians to CMVD and promote basic and clinical research on CMVD in TCM.

## 2 Clinical significance of CMVD

At present, there are no large-scale epidemiological data for CMVD, and its incidence and prognostic significance have not been clear. A study of predictive models of coronary artery disease in patients with chest pain showed that up to 70% of angina patients had no obstructive coronary stenosis, as observed by coronary angiography. In most of these patients, the coronary angiography results were normal or nearly normal (Reeh et al., 2019). Recent studies have shown that CMVD is an independent predictor of adverse cardiovascular events, and there are significant associations with the incidence of cardiovascular events such as ejection fraction (EF) reserved heart failure, myocardial ischemia, acute coronary syndrome, myocardial infarction, stroke, and cardiogenic death (Gulati et al., 2009; Lind et al., 2011; Jespersen et al., 2012; Crea et al., 2014; Maddox et al., 2014; Petersen et al., 2014; Brainin et al., 2018; Herscovici et al., 2018). In people with chest pain and normal coronary angiography results, the incidence of CMVD was as high as 45%–60%, and the incidence and mortality due to myocardial ischemia, angina, myocardial infarction, and other cardiovascular events were significantly increased (Crea et al., 2014). A 7.5-year follow-up study of 11,223 patients with stable angina showed that nearly one-third of the men and two-thirds of the women admitted to the hospital had no coronary artery disease, and both men and women had significantly higher rates of major cardiovascular events and all-cause mortality in patients with normal and nonobstructive coronary artery disease than in the controls. Researchers speculate that CMVD may be an important cause of poor prognoses in these patients (Jespersen et al., 2012).

Coronary flow reserve (CFR), first proposed by Gould in 1974, is an important physiological measure of coronary microcirculation and can be used to evaluate the reserve function of both subepicardial coronary arteries and microcirculation (Gould et al., 1974). In CMVD without obstructive coronary artery disease, a decrease in CFR is a marker of coronary microcirculatory disturbance (Gould, 2009). At present, there is no consensus on the optimal CFR cut-off value for diagnosing CMVD. CFR values <2.0 are generally recommended as the critical value for diagnosing coronary microvascular dysfunction (Murthy et al., 2014), whereas CFR values <2.5 indicate coronary microvascular disease (Driessen et al., 2017). Decreases in CFR were associated with adverse cardiovascular events (Murthy et al., 2012; Murthy et al., 2014). Decreased CFRs (<1.6) in women were found to predict higher incidences of adverse cardiovascular events (Taqueti et al., 2017). Taqueti et al. (2018) found that patients with nonocclusive coronary artery stenosis who had chest pain and normal left ventricular EFs appeared to be at low risk of heart failure, and a decrease in CFR could lead to an increase in the number of adverse cardiac events. The poor prognosis and pathogenesis of heart failure with preserved EF (HFpEF) are closely related to CMVD. Gdowski et al. published a meta-analysis of 6,631 patients with suspected myocardial ischemia but without coronary artery occlusion, which showed that compared to patients with normal CFRs, CFR decreased the mortality of patients by 3.93 times, whereas the incidence of adverse cardiovascular events increased by 5.16 times (Gdowski et al., 2020). Because CMVD is common in patients with cardiovascular risk factors, CFR measurements should be performed early to determine the presence of CMVD even in people without coronary artery disease to help with early diagnosis and intervention to prevent further progression of CMVD and adverse cardiac events (Del Buono et al., 2021; Tjoe et al., 2021; Lopez et al., 2022). CMVD has a complex etiology, involves a wide range of people, and lacks standardized and effective means of detection, which causes the systematic prevention and management of coronary heart disease to be difficult and seriously affects the prognosis of patients. Therefore, the early diagnosis and treatment of CMVD have important clinical significance (Taqueti et al., 2018; Bairey Merz et al., 2020).

## 3 Pathogenesis of CMVD

At present, the pathogenesis of CMVD is not completely clear and is mainly divided into structural and functional abnormalities of coronary microvessels.

### 3.1 Coronary microvascular structural abnormalities

Abnormal structures of the coronary artery microvasculature are closely related to increases in left ventricular mass (Huo and Kassab, 2012), which is common in hypertrophic cardiomyopathy and hypertension (Camici et al., 2020); it is often accompanied by intimal thickening, which results in a mild reduction in the lumen area of the arterioles (Taqueti et al., 2018; Camici et al., 2020). Arteriosclerosis can exacerbate this change and lead to microvascular occlusion, narrowing of intramural arterioles and capillary lumen, and capillary thinning (Labazi and Trask, 2017).

### 3.2 Coronary microvascular dysfunction

Coronary microvascular dysfunction (CMD) includes endothelial cell-dependent vascular abnormalities, which are commonly seen in people with diabetes, obesity, smoking, and other cardiovascular risk factors, and dependent vasodilation abnormalities, microvascular spasms, microvascular embolisms, and extravascular mechanisms (Zhang et al., 2017; Ford et al., 2018). These physiological and pathological changes in the development of CMD play roles to varying degrees.

During CMVD development, injury to coronary microvascular endothelial cells (CMECs) is the key link (Yin et al., 2021). Current studies suggest that angina in patients with CMVD is associated with myocardial ischemia, and a CMD-induced decrease in CFR is thought to be the main cause of CMVD (Sucato et al., 2017; Padro et al., 2020). CMEC accounts for approximately 1/3 of the total number of cardiac cells and plays an important role in maintaining the normal function of coronary microvessels (Li et al., 2001). CMEC dysfunction often precedes myocardial injury (Scarabelli et al., 2001). Related studies show that the normal proliferation, adhesion, migration, apoptosis, and secretion of CMECs are impaired by risk factors such as old age (Moreau et al., 1998), hypertension (Rizzoni et al., 2003), hyperlipidemia (Kaufmann et al., 2000b), smoking (Kaufmann et al., 2000a), obesity (Chen et al., 2017), insulin resistance (Dagres et al., 2004), and diabetes mellitus (Pitkanen et al., 1998), which leads to CMD, decreased CFRs, insufficiency of myocardial blood supply, and finally occurrence of CMVD (Baumgart et al., 1999; Camici and Crea, 2007).

Microvascular spasms are one of the important mechanisms of CMVD. In some patients with atypical angina but with normal coronary angiography results, abnormal activation of the myocardial α2 adrenergic receptor at rest due to sympathetic nervous system dysfunction leads to coronary microvascular contraction and reduced myocardial perfusion, which cause myocardial ischemia (Baumgart et al., 1999). Microvascular embolization is also an important mechanism of CMVD. Due to interventional therapy or plaque rupture, microthrombi, atheromatous plaque fragments, and thromboemboli block the blood flow in microvessels, which leads to distal microvascular occlusion and causes CMVD (Crea et al., 2014). The extravascular mechanism can be seen in diseases with significantly elevated left ventricular diastolic pressures, such as left ventricular hypertrophy and left ventricular fibrosis, and diseases that can directly decrease coronary diastolic pressures, such as aortic stenosis, severe coronary stenosis, anterior arteriole stenosis, and hypotension (Giacco and Brownlee, 2010). In addition, the formation and development of CMVD is a cumulative process in which metabolic disorders, oxidative stress, and inflammatory reactions play important roles (Labazi and Trask, 2017; Long et al., 2017; Zhang et al., 2017; Oikonomou et al., 2018; Du et al., 2019).

## 4 Modern drug therapy of CMVD

### 4.1 Classic anti-myocardial ischemia drugs

Beta-blockers and short-acting nitrates are first-line drugs used to control the symptoms of CMVD (Task Force et al., 2013). Patients who cannot tolerate beta-blockers can be treated with ivabradine instead (Mumma and Flacke, 2015). In the absence of adequate symptom control, the use of calcium antagonists and/or long-acting nitrates in addition to beta-blockers may contribute to improved control of patient symptoms (Task Force et al., 2013). RAAS inhibitors (including ACEIs and ARBs) can also improve coronary artery microvascular function by blocking vasoconstriction due to angiotensinogen II (Task Force et al., 2013). Coronary revascularization is a reasonable approach for CMVD patients with epicardial coronary artery occlusion and may improve the clinical symptoms and prognosis of CMVD patients (Task Force et al., 2013; Taqueti et al., 2015). For patients with cardiogenic chest pain who show signs of ischemia in perfusion tests, beta-blockers reduce myocardial oxygen consumption and improve symptoms. However, in patients with variant angina, beta-blockers should be avoided, and calcium channel blockers should be used as first-line drugs (Padro et al., 2020).

### 4.2 Non-classic anti-myocardial ischemia drugs

A variety of nonclassical antianginal agents have been used in CMVD patients, including nicorandil, ivabradine, trimetazidine, and ranolazine (Villano et al., 2013; Cattaneo et al., 2015; Bairey Merz et al., 2016; Shah et al., 2017). Nicorandil is one of the most studied drugs, and some studies have shown that nicorandil can effectively improve exercise-induced myocardial ischemia without altering cardiac autonomic nerve activity. It is suggested that nicorandil may have a direct vasodilative effect on coronary microvessels in patients with CMVD (Lanza et al., 2014). In addition, studies have shown that nicorandil can improve the symptoms of CMVD patients by inhibiting inflammatory factors and improving vascular endothelial function (He et al., 2017).

### 4.3 Other drugs

Statins are commonly used in the treatment of CMVD. In addition to lowering cholesterol levels, statins inhibit vascular inflammation, increase eNOS levels, and increase NO availability in blood vessels (Padro et al., 2020). Data analysis for eight thrombolytics in myocardial infarction (TIMI) trials found lower 30-day mortalities or reinfarction rates in patients with non-ST-segment elevation acute coronary syndromes (NSTE-ACS) and nonobstructive CAD who received statins (De Ferrari et al., 2014). In addition, long-term follow-up data from the Swedish Web-system for enhancement and development of evidence-based care in heart disease evaluated according to recommended therapy study confirmed that statins can decrease the number of major cardiovascular events (e.g., all-cause mortality, myocardial infarction, ischemic stroke, and heart failure) in patients with CMVD (Lindahl et al., 2017). Statins and ACE inhibitors are recommended for patients with cardiovascular risk factors associated with arteriosclerosis or endothelial dysfunction (Padro et al., 2020). Long-term l-arginine supplementation can improve vascular endothelial function, coronary flow, and symptoms in patients with CMVD (Lerman et al., 1998).

The treatment goals for CMVD include minimizing angina attacks and their associated symptoms, maximizing improvements in patients’ physical functions and quality of life, and extending their lifespans. The treatment options available to patients are very limited due to a lack of standardized diagnoses, nonstandard clinical designs, small sample sizes, and insufficient evidence for clinical improvement of CMVD. At present, most of the drug therapies used for CMVD are empirically based. Although they have certain clinical effects, many patients are still repeatedly hospitalized for chest pain and coronary angiography studies, which seriously affect the quality of life of patients (Zhuang et al., 2020). Modern medicine lacks a definitive treatment for CMVD (Taqueti and Di Carli, 2018).

A clinical study indicated that CMVD treatments by integrating traditional Chinese and Western medicine (WM) were superior to those using Western medicine alone (Ji and Ren, 2009; Li and Yu, 2010; Sun and Wu, 2012; Wu et al., 2013; Chen and Ma, 2014; Lv et al., 2014; Wang et al., 2014; Luo et al., 2017; Qin et al., 2017; Wei, 2018; Yan et al., 2018; Zhang, 2019; Qiu et al., 2021; Sun, 2021; Zhang, 2021; Cao et al., 2022; Chen et al., 2022). A systematic review and meta-analysis of randomized controlled trials (RCTs) involving 1,903 patients with CMVD revealed that integrated Chinese and Western medicine therapies were more effective than Western medicine treatments alone regarding the indicators of frequency of angina pectoris attacks, electrocardiography (ECG), nitroglycerin amounts needed, treadmill exercise tests, TCM syndrome scores, and levels of C-reactive protein (CRP), endothelin-1 (ET-1), and nitric oxide (NO) (Zhong et al., 2020). Another systematic review and meta-analysis of RCTs that included 1,075 CMVD patients showed consistent results that compared to Western medicine treatments alone, TCM with WM treatment could further increase CFRs, decrease the index of microvascular resistance (IMR), increase NO levels, and decrease levels of high-sensitivity C-reactive protein (hs-CRP) (Wang M. X. et al., 2021).

## 5 TCM in the treatment of CMVD

### 5.1 Understanding CMVD in TCM theory

There is no specific record of CMVD in ancient Chinese literature. In view of the typical symptoms of angina pectoris, in traditional Chinese medicine, most doctors classify it as “Xiong Bi” and “Xin Tong.” However, considering the microvascular anatomy of coronary arteries, some doctors think that CMVD should belong to the “Luo Bing” described in TCM (Chang et al., 2016). The etiology and pathogenesis of CMVD in TCM have not been universally recognized, but most doctors believe that CMVD is mainly caused by emotional discomfort, stagnation of liver-qi, and the interaction of phlegm and blood stasis in the chest (Bi et al., 2013).

### 5.2 Clinical evidence of TCM for CMVD

**FIGURE 2:**
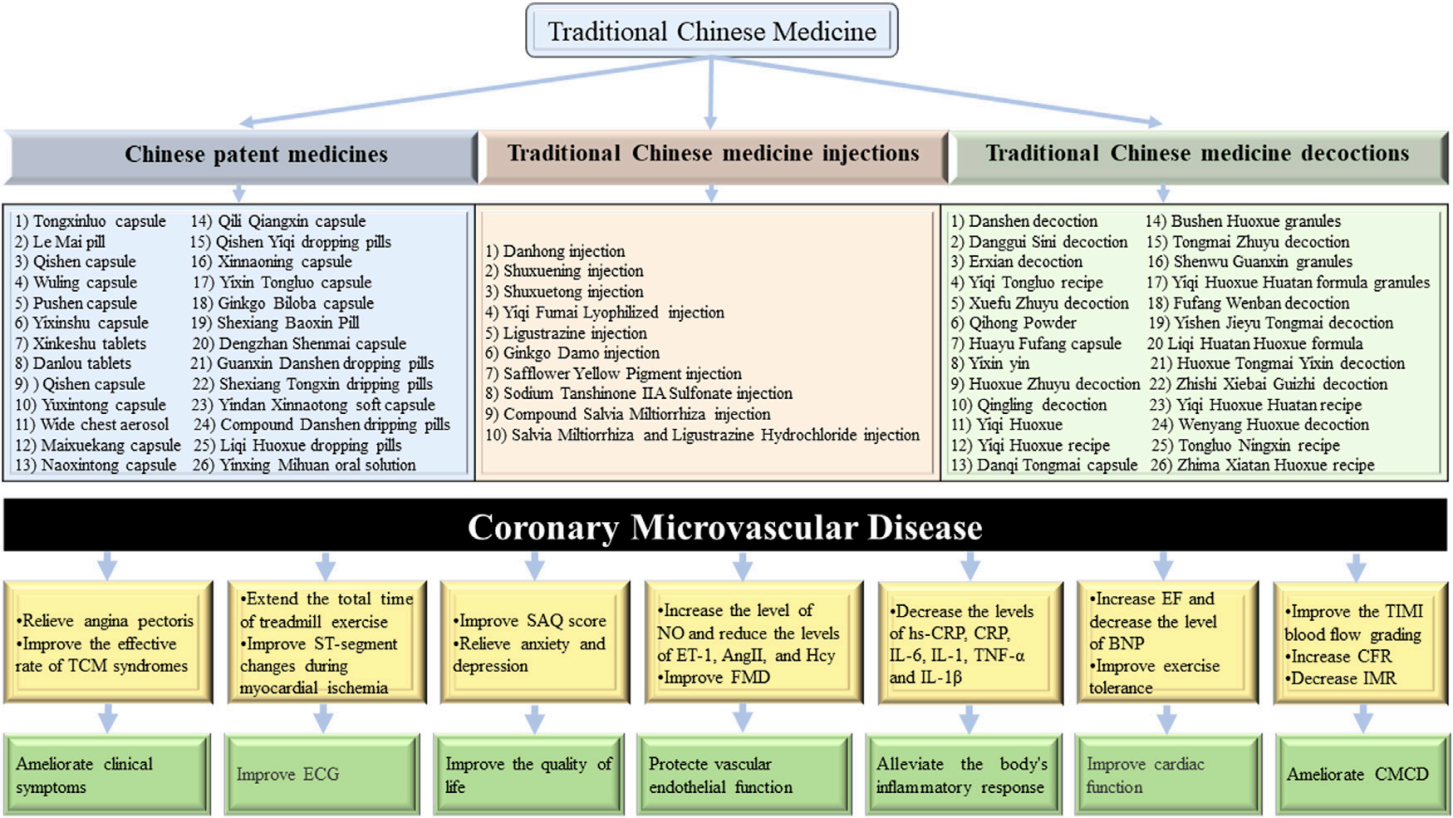
Clinical evidence of TCM for CMVD.

A total of 71 representative RCTs, including 34 Chinese patent medicines (CPMs), 11 TCM injections, and 26 TCM decoctions, were compiled and summarized. According to the available clinical data, there have been many clinical trials on the clinical advantages of TCM in the prevention and treatment of CMVD, as shown in Figure 2. The clinical evidence of TCM for CMVD, including the outcomes of 1) clinical manifestations, 2) coronary microcirculation, and 3) laboratory results, are analyzed and summarized in Table 1. Based on Table 1, the clinical evidence of TCM for the typical manifestations of CMVD is summarized in Table 2.

**TABLE 1:** Clinical evidence of TCM for CMVD.

No.	Location	Name of traditional Chinese medicine	Compositions	Duration of treatment	Intervention group *vs*. control group	Sample size (T/C)	Main clinical symptoms	Laboratory finding	Reference
1	Liaoning Provincial People’s Hospital	Tongxinluo capsule (TXL)	*Paeonia anomala* subsp. *veitchii* (Lynch) D.Y.Hong & K.Y.Pan (Chishao), *Dalbergia odorifera* T.C.Chen (Jiangxiang), *Boswellia sacra* Flück. (Ruxiang), Periostracum Cicadae (Chantui), Scorpio (Quanxie), Eupolyphaga Steleophaga (Tubiechong), *Panax ginseng* C.A.Mey. (Renshen), *Cyperus brevifolius* (Rottb.) Hassk. (Wugong), *Hirudo* (Shuizhi), *Santalum album* L. (Tanxiang), *Ziziphus jujuba* Mill. (Suanzaoren), Borneolum Syntheticum (Bingpian)	3 months	(RCT) TXL + CT *vs*. CT	60 (29/31)	① I 93.10% *vs*. C 87.10% (*p* < 0.05) ② I 86.21% *vs*. C 77.42% (*p* < 0.05)	Unreported	Liu et al. (2013)
2	Ezhou Central Hospital of Hubei Province	Tongxinluo capsule (TXL)	—	3 months	(RCT) TXL + CT *vs*. CT	68(34/34)	① I 82.35% *vs*. C 58.82% (*p* < 0.05) ④The total exercise time: I 9.40 ± 1.60 *vs*. C 7.35 ± 1.25 (*p* < 0.05); the time when the ST segment was depressed by 1 mV I 6.85 ± 6.42 *vs*. C 5.21 ± 1.36 (*p* < 0.05); the ST segment depression I 0.60 ± 0.18 *vs*. C 0.86 ± 0.32 (*p* < 0.05)	③Increase the level of NO and decrease the levels of ET-1 and ET-1/NO (*p* < 0.05)	Wang. (2016)
3	Huangshi Central Hospital	Tongxinluo capsule (TXL)	—	8 weeks	(RCT) TXL + CT *vs*. CT	39(19/20)	① I 89.5% *vs*. C 50.0% (*p* < 0.05) ② I 78.9% *vs*. C 45.0% (*p* < 0.05)	③Decrease the level of vWF (*p* < 0.05)	Fang et al. (2018)
4	The First Hospital of Fangshan District of Beijing	Shexiang Baoxin pill (SXBX)	*Moschus* (Shexiang), *Panax ginseng* C.A.Mey*.* (Renshen), *Calculus Bovis* (Niuhuang), *Neolitsea cassia* (L*.*) *Kosterm.* (Rougui), *Liquidambar orientalis* Mill*.* (Suhexiang), *Venenum Bufonis* (Chansu), *Borneolum Syntheticum* (Bingpian)	6 weeks	(RCT) SXBX + CT vs. CT	56 (28/28)	① I 92.86% *vs*. C 71.43% (*p* < 0.05) ⑦ I 1.2 ± 0.8 *vs*. C 1.9 ± 0.9 (*p* < 0.05)	⑧Reduce the levels of TC, TG, and LDL-C and increase the level of HDL-C (*p* < 0.05)	Zhang et al. (2013a)
5	The First People’s Hospital of Fuzhou	Shexiang Baoxin pill (SXBX)	—	3 months	(RCT) SXBX + CT *vs*. CT	76 (38/38)	① I 97.37% *vs*. C 76.32% (*p* < 0.05) ②The improvement in ECG was more obvious in the intervention group (*p* < 0.05)	③Increase the level of NO and reduce the level of ET-1 (*p* < 0.05) ⑤Decrease the levels of hs-CRP, IL-1, and IL-6 (*p* < 0.05)	Wu et al. (2019)
6	The Affiliated Hospital of Shanxi University of Traditional Chinese Medicine	Shexiang Baoxin pill (SXBX)	—	3 months	(RCT) SXBX + CT *vs*. CT	100 (50/50)	⑬ I 94.0% *vs*. C 78.0% (*p* < 0.05)	③Increase the level of NO and reduce the level of ET-1 (*p* < 0.05) ⑤Decrease the level of hs-CRP (*p* < 0.05)	Jin. (2018)
7	Chengdu Military General Hospital	Yindan Xinnaotong soft capsule (YDXNT)	*Ginkgo biloba* L. (Yinxingye), *Salvia miltiorrhiza* Bunge (Danshen), *Erigeron breviscapus* (Vaniot) Hand.-Mazz. (Dengzhanxixin), *Codonopsis pilosula* (Franch.) Nannf. (Jiaogulan), *Allium sativum* L*.* (Dasuan), *Crataegus pinnatifida* Bunge (Shanzha), *Panax notoginseng (Burkill)* F.H.Chen (Sanqi), *Blumea balsamifera (*L.) DC. (Aipian)	6 months	(RCT) YDXNT + CT *vs*. CT	87 (43/44)	①Ameliorate angina pectoris	③Increase the level of NO and reduce the level of ET-1 (*p* < 0.05) ⑤Decrease the level of hs-CRP (*p* < 0.05)	Wang et al. (2019)
8	Ninth People’s Hospital of Zhengzhou	Yindan Xinnaotong soft capsule (YDXNT)	—	6 months	(RCT) YDXNT + CT *vs*. CT	130 (65/65)	① I 95.38% vs. C 83.08% (*p* < 0.05)	③Increase the levels of NO and VEGF and reduce the level of ET-1 (*p* < 0.05) ⑤Reduce the levels of CRP, IL-1, and IL-6 (*p* < 0.05)	Wang and Li. (2022)
9	Affiliated Hospital to Shanxi University of Chinese Medicine	Bushen Huoxue Granules (BSHX)	*Epimedium sagittatum (Siebold & Zucc.) Maxim.* (Yinyanghuo), *Curculigo orchioides Gaertn.* (Xianmao), *Panax notoginseng (Burkill) F.H.Chen* (Sanqi), *Conioselinum anthriscoides “Chuanxiong”* (Chuanxiong), *Santalum album L.* (Tanxiang)	4 months	(RCT) BSHX + CT *vs*. CT	64 (43/25)	① I 90.69% *vs*. C 80.00% (*p* > 0.05) ② I 86.05% vs*.* C 60.00% (*p* < 0.05)	③Improve the level of NO and reduce the level of ET-1 (*p* < 0.05)	Wen et al. (2016)
10	The Third Hospital of Wuhan	Compound Danshen Dripping pills (CDDP)	*Salvia miltiorrhiza* Bunge (Danshen), *Panax notoginseng* (Burkill) F.H.Chen (Sanqi), Borneolum Syntheticum (Bingpian)	3 months	(RCT) CDDP + CT *vs*. CT	124 (62/62)	①Angina pectoris attack times: I 4.58 ± 0.84 *vs*. C 2.09 ± 0.44 (*p* < 0.05)	⑪Improved Chinese medical syndrome (*p* < 0.05) ⑩Improve coronary blood flow (*p* < 0.05)	Zhang and Chen. (2020)
11	The Third Affiliated Hospital of Shandong First Medical University	Compound *Salvia miltiorrhiza* injection (CSMI)	*Salvia miltiorrhiza* Bunge (Danshen), *Dalbergia odorifera* T.C.Chen (Jiangxiang)	14 days	(RCT) CSMI + CT *vs*. CT	106 (53/53)	Unreported	③Improve the level of NO and reduce the level of ET-1 (*p* < 0.05); improve FMD (*p* < 0.05) ⑤Decrease the levels of CRP, IL-6, and TNF-α (*p* < 0.05) ⑨Decrease the levels of plasma viscosity, fibrinogen, and erythrocyte aggregation index (*p* < 0.05)	Liu and Gu. (2021)
12	The First Affiliated Hospital of Heilongjiang University of Chinese Medicine	Danggui Sini decoction (DGSN)	*Angelica sinensis* (Oliv.) Diels (Danggui), *Cinnamomum cassia* (L.) J. Presl (Guizhi), *Asarum sieboldii* Miq. (Xixin), *Equisetum hyemale* L*.* (Tongcao), *Paeonia lactiflora* Pall*.* (Baishao), *Ziziphus jujuba* Mill*.* (Dazao), *Glycyrrhiza uralensis* Fisch. ex DC. (Zhigancao)	3 weeks	(RCT) DGSN + CT *vs*. CT	68 (34/34)	① I 85.29% *vs*. C 67.65% (*p* < 0.05); angina pectoris attack times: I 2.01 ± 1.88 *vs*. C 7.69 ± 1.76 (*p* < 0.05); angina pectoris pain score: I 0.89 ± 1.23 *vs*. C 3.45 ± 1.34 (*p* 0.05); angina pectoris duration: I 1.65 ± 1.72 *vs*. C 3.78 ± 1.69, *p*0.05 ②Improved ECG	Unreported	Cui et al. (2021)
13	The Fourth Affiliated Hospital of Harbin Medical University	Danhong injection (DH)	*Salvia miltiorrhiza* Bunge (Danshen), *Carthamus tinctorius* L*.* (Honghua)	10 days	(RCT) DH + CT *vs*. CT	60 (30/30)	① I 93.33% *vs*. C 73.33% (*p* < 0.05) ② I 83.33% *vs*. C 56.67% (*p* < 0.05) ④ The total exercise time: I 486.9 ± 83.0 *vs*. C 438.3 ± 85.6 (*p* < 0.05); the time when the ST segment was depressed by 1 mm: I 351.9 ± 69.2 *vs*. C 310.7 ± 79.2 (*p* < 0.05)	③Decrease the levels of ET-1 and TM (*p* < 0.05) ⑤Reduce the level of E-selectin (*p* < 0.05)	Hu et al. (2014)
14	East Branch of Shanghai Sixth People’s Hospital	Danhong injection (DH)	—	2 weeks	(RCT) DH + CT *vs*. CT	92 (47/45)	① I 78.7% *vs*. C 55.6% (*p* < 0.05)	⑤Decrease the level of hs-CRP (*p* < 0.05)	Chen et al. (2018)
15	Mingdong Hospital	Danlou tablets (DLT)	*Trichosanthes kirilowii* Maxim*.* (Gualoupi), *Allium chinense* G.Don (Xiebai), *Pueraria montana* var. *lobata* (Willd.) Maesen & S.M.Almeida ex Sanjappa & Predeep (Gegen), *Conioselinum anthriscoides* “*Chuanxiong*” (Chuanxiong), *Salvia miltiorrhiza* Bunge (Danshen), *Paeonia anomala* subsp. *veitchii* (Lynch) D.Y.Hong & K.Y.Pan (Chishao), *Alisma plantago-aquatica* subsp. *orientale* (Sam.) *Sam.* (Zexie), *Astragalus mongholicus* Bunge (Huangqi), *Drynaria roosii Nakaike* (Gusuibu), *Curcuma aromatica Salisb.* (Yujin)	12 weeks	(RCT) DLT + CT *vs*. CT	80 (40/40)	① I 80.0% *vs*. C 52.5% (*p* < 0.05) ② I 80.0% *vs*. C 57.5% (*p* < 0.05)	⑭Improve EF (*p* < 0.05)	Zheng et al. (2019)bib_Zheng_et_al_2019
16	Chang’an District Hospital of the First Affiliated Hospital of Xi’an Jiaotong University	Danqi Tongmai capsule (DTC)	*Salvia miltiorrhiza* Bunge (Danshen), *Astragalus mongholicus* Bunge (Huangqi), *Carthamus tinctorius* L*.* (Honghua), *Conioselinum anthriscoides* “*Chuanxiong*” (Chuanxiong), *Panax notoginseng* (Burkill) F.H.Chen (Sanqi), *Juglans regia* L. (Taoren), *Codonopsis pilosula* (Franch.) Nannf. (Dangshen)	4 weeks	(RCT) DTC + CT *vs*. CT	84 (42/42)	Unreported	③Reduce the levels of ET-1 and Ang II (*p* < 0.05) ⑩Improve the TIMI classification ⑭Decrease the LVEDd (*p* < 0.05)	Ren et al. (2019)
17	The Second Affiliated Hospital of Heilongjiang University of Chinese Medicine	Danshen decoction (DSD)	*Salvia miltiorrhiza* Bunge (Danshen), *Santalum* album L*.* (Tanxiang), *Wurfbainia villosa* var*. xanthioides* (Wall. ex Baker) Skornick. & A.D.Poulsen (Sharen)	4 weeks	(RCT) DSD + CT *vs*. CT	60 (30/30)	① I 93.3% *vs*. C 83.3% (*p* < 0.05) ② I 90.0% *vs*. C 76.7% (*p* < 0.05)	Unreported	(Chen et al. (2021)
18	The Third Hospital of Hebei Medical University	Dengzhan Shenmai capsule (DSC)	*Erigeron breviscapus* (Vaniot) Hand.-Mazz. (Dengzhanxixin), *Panax ginseng* C.A.Mey. (Renshen), *Schisandra chinensis* (Turcz.) Baill*.* (Wuweizi), *Ophiopogon japonicus* (Thunb.) *Ker Gawl.* (Maidong)	6 months	(RCT) DSC + CT *vs*. CT	120 (60/60)	⑬I 65.0% *vs*. C 41.67% (*p* < 0.05)	③Improve the level of NO and reduce the levels of ET-1 and APN (*p* < 0.05) ⑤Decrease the level of hs-CRP (*p* < 0.05) ⑭Improve the cardiac function	Liu et al. (2017)
19	Xijing Hospital, the Fourth Military Medical University	Erxian decoction (ED)	*Curculigo orchioides Gaertn.* (Xianmao), *Epimedium sagittatum* (Siebold & Zucc.) Maxim. (Yinyanghuo), *Gynochthodes officinalis* (F.C.How) Razafim. & B.Bremer (Bajitian), *Angelica sinensis* (Oliv.) *Diels* (Danggui), *Phellodendron amurense* Rupr. (Huangbo), *Anemarrhena asphodeloides* Bunge (Zhimu)	4 weeks	(RCT) ED + CT *vs*. CT	80(30/50)	④The total exercise time: I 685.0 ± 30.3 *vs*. C 672.2 ± 24.1 (*p* < 0.05); the time when the ST segment was depressed by 1 mm I 323.8 ± 11.41 *vs*. C 443.0 ± 27.1 (*p* < 0.05)	Unreported	Zhang et al. (2012)
20	Tongzhou District Hospital of Traditional Chinese Medicine	Fufang Wenban decoction (FFWB)	*Cyperus brevifolius* (Rottb.) Hassk. (Wugong), *Pinellia ternata* (Thunb.) *Makino* (Banxia), *Hirudo* (Shuizhi), *Atractylodes macrocephala* Koidz. (Baizhu), *Scorpio* (Quanxie), *Citrus aurantium* L*.* (Chenpi), *Pheretima* (Dilong)	1 month	(RCT) FFWB + CT *vs*. CT	86 (43/43)	① I 93.02% *vs*. C 79.07% (*p* < 0.05)	③Improve the level of NO and reduce the level of ET-1 (*p* < 0.05) ⑤Decrease the level of hs-CRP (*p* < 0.05)	Jiang. (2021)
21	Huizhou Hospital of Traditional Chinese Medicine	Ginkgo Biloba capsule (GBC)	*Ginkgo biloba* ***L.*** (Yinxingye)	30 days	(RCT) GBC + CT *vs*. CT	80 (40/40)	① I 92.50% *vs*. C 75.00% (*p* < 0.05) ② I 72.50% *vs*. C 50.00% (*p* < 0.05)	⑧Reduce the levels of TG, TC, and LDL-C and increase the level of HDL-C (*p* < 0.05)	Zou et al. (2016)
22	Harbin Medical Sciences University	Ginkgo Damo injection (GDI)	*Ginkgo biloba* L*.* (Yinxingye)	3 months	(RCT) GDI + CT *vs*. CT	60 (30/30)	① I 86.7% *vs*. C 50.0% (*p* < 0.05) ② I 60.0% *vs*. C 40.0% (*p* < 0.05)	⑩Improve coronary blood flow (*p* < 0.05)	Wang et al. (2010)
23	Wuhan No. 1 Hospital	Guanxin Danshen Drop pills (GDDP)	*Salvia miltiorrhiza* Bunge (Danshen), *Panax notoginseng* (Burkill) F.H.Chen (Sanqi), *Dalbergia odorifera* T.C.Chen (Jiangxiang)	6 months	(RCT) GDDP + CT *vs*. CT	30 (15/15)	⑬I 80.0% *vs*. C 46.7% (*p* < 0.05)	⑩Improve coronary blood flow (*p* < 0.05)	Fang et al. (2013)
24	Zaozhuang Municipal Hospital	Guanxin Shutong capsule (GSC)	*Choerospondias axillaris* (Roxb.) B.L.Burtt & A.W.Hill (Guangzao), *Salvia miltiorrhiza* Bunge (Danshen), *Syzygium aromaticum* (L.) Merr. & L.M.Perry (Dingxiang), *Borneolum Syntheticum* (Bingpian), *Bambusa textilis* McClure (Tianzhuhuang)	6 months	(RCT) GSC + CT *vs*. CT	40 (21/19)	① I 76.2% *vs*. C 52.6% (*p* < 0.05) ② I 52.40% *vs*. C 47.40% (*p* < 0.05)	⑩Improve coronary blood flow (*p* < 0.05)	Li. (2014)
25	Nantong Traditional Chinese Medicine Hospital	Huayu Fufang capsule (HYFF)	*Hirudo* (Shuizhi), *Panax notoginseng* (Burkill) F.H.Chen (Sanqi), *Eupolyphaga Steleophaga* (Tubiechong)	2 months	(RCT) HYFF + CT *vs*. CT	62 (27/35)	① I 88.6% *vs*. C 66.7% (*p* < 0.05) ④The ST segment depression I 0.80 ± 0.25 *vs*. C 1.0 ± 0.26 (*p* < 0.05)	③Increase the level of NO and reduce the level of ET-1 (*p* < 0.05) ⑤Decrease the level of hs-CRP (*p* < 0.05)	Wu et al. (2010)
26	Xiantao First People’s Hospital Affiliated to Yangtze University	Huoxue Tongmai Yixin decoction (HTY)	*Astragalus mongholicus* Bunge (Huangqi), *Codonopsis pilosula* (Franch.) Nannf. (Dangshen), *Conioselinum anthriscoides “Chuanxiong”* (Chuanxiong), *Salvia miltiorrhiza* Bunge (Danshen), *Ophiopogon japonicus* (Thunb.) Ker Gawl. (Maidong), *Carthamus tinctorius* L*.* (Honghua), *Polygala tenuifolia* Willd*.* (Yuanzhi), *Wurfbainia villosa* var. *xanthioides* (Wall. ex Baker) Skornick. & A.D.Poulsen (Sharen), *Poria cocos* (Schw.) *Wolf* (Fuling), *Glycyrrhiza uralensis* Fisch. ex DC. (Gancao), *Curcuma aromatica* Salisb. (Yujin)	4 weeks	(RCT) HTY + CT *vs*. CT	140 (70/70)	①Angina pectoris attack times: I 2.70 ± 0.62 *vs*. C 4.25 ± 0.96 (*p* < 0.05); angina pectoris pain score: I 2.61 ± 0.33 *vs*. C 3.50 ± 0.58 (*p* < 0.05); angina pectoris duration: I 4.04 ± 0.87 *vs*. C 8.93 ± 1.53 (*p* < 0.05) ④Improve treadmill exercise test ⑪ I 94.29% *vs*. C 81.43% (*p* < 0.05) ⑫ I 4.5 ± 0.35 *vs*. C 3.46 ± 0.21 (*p* < 0.05)	③Improve the level of NO and reduce the level of ET-1 (*p* < 0.05) ⑤Decrease the level of hs-CRP (*p* < 0.05)	Mao et al. (2021)
27	The First Affiliated Hospital of Henan University	Huoxue Zhuyu decoction (HXZY)	*Astragalus mongholicus* Bunge (Huangqi), *Codonopsis pilosula* (Franch.) Nannf. (Dangshen), *Conioselinum anthriscoides "Chuanxiong”* (Chuanxiong), *Juglans regia* L*.* (Taoren), *Angelica sinensis* (Oliv.) Diels (Danggui), *Carthamus tinctorius* L*.* (Honghua), *Cornus officinalis* Siebold & Zucc. (Shanzhuyu), *Rehmannia glutinosa* (Gaertn.) DC. (Dihuang), *Bupleurum chinense* DC*.* (Chaihu), *Glycyrrhiza uralensis* Fisch. ex DC. (Gancao)	12 weeks	(RCT) HXZY + CT *vs*. CT	88 (44/44)	①Angina pectoris attack times: I 3.75 ± 0.93 *vs*. C 5.01 ± 1.28 (*p* < 0.05); angina pectoris pain score: I 1.27 ± 0.40 *vs*. C 2.13 ± 0.52 (*p* < 0.05); angina pectoris duration: I 2.23 ± 0.82 *vs*. C 3.34 ± 1.16 (*p* < 0.05)⑪ I 3.46 ± 0.83 *vs*. C 7.69 ± 1.52 (*p* < 0.05) ⑬ I 94.45% *vs*. C 81.82% (*p* < 0.05)	③Improve the level of NO and reduce the level of ET-1 (*p* < 0.05) ⑤Decrease the level of hs-CRP (*p* < 0.05)	Guo et al. (2020)
28	Shaanxi Provincial Hospital of Chinese Medicine	Le Mai pill (LMP)	*Salvia miltiorrhiza* Bunge (Danshen), *Conioselinum anthriscoides “Chuanxiong”* (Chuanxiong), *Paeonia anomala* subsp*. veitchii* (Lynch) D.Y.Hong & K.Y.Pan (Chishao), *Carthamus tinctorius* L*.* (Honghua), *Dolomiaea costus* (Falc.) Kasana & A.K.Pandey (Muxiang), *Cyperus rotundus* L*. (Xiangfu)*, *Crataegus pinnatifida* Bunge (Shanzha)	6 weeks	(RCT) LMP + CT *vs*. CT	87 (45/42)	① I 66.7% *vs*. C 50.0% (*p* < 0.05) ② I 55.6% *vs*. C 40.5% (*p* < 0.05)	Unreported	Zhang and Xie. (2020)
29	Affiliates Hospital of Shaanxi University of Chinese Medicine	Ligustrazine injection (LI)	*Conioselinum anthriscoides “Chuanxiong”* (Chuanxiong)	7 days	(RCT) LI + CT *vs*. CT	60 (30/30)	① I 96.7% *vs*. C 83.3% (*p* < 0.05)	③Improve FMD (*p* < 0.05) ⑤Decrease the levels of CRP and IL-6 (*p* < 0.05)	Yue et al. (2018)
30	First Teaching Hospital of Tianjin University of Traditional Chinese Medicine	Liqi Huatan Huoxue Formula (LHHF)	*Bupleurum chinense* DC. (Chaihu), *Citrus aurantium* L. (Zhiqiao), *Trichosanthes kirilowii* Maxim*.* (Gualoupi), *Allium chinense* G.Don (Xiebai), *Poria cocos* (Schw.) *Wolf* (Fuling), *Atractylodes macrocephala Koidz.* (Baizhu), *Citrus aurantium* L*.* (Chenpi), *Conioselinum anthriscoides* “*Chuanxiong*” (Chuanxiong), *Angelica sinensis* (Oliv.) Diels (Danggui), *Salvia miltiorrhiza* Bunge (Danshen), *Corydalis yanhusuo* (Y.H.Chou & Chun C.Hsu) W.T.Wang ex Z.Y.Su & C.Y.Wu (Yanhusuo), *Carthamus tinctorius* L*.* (Honghua)	2 weeks	(RCT) LHHF + CT *vs*. CT	55 (28/27)	① I 82.14% *vs*. C 55.56% (*p* < 0.05); ④ the maximum metabolic equivalents: I 7.24 ± 0.87 *vs*. C 6.21 ± 0.72 (*p* < 0.05); exercise time prolonged: I 346.54 ± 71.66 *vs*. C 295 ± 65.73 (*p* < 0.05); the sun of ST segments depression decreased I: 5.3 ± 1.8 *vs*. C 6.2 ± 1.7(*p* < 0.05) ⑪ I 85.71% *vs*. C 55.56% (*p* < 0.05)	③Increase the level of NO and reduce the level of ET-1 (*p* < 0.05) ⑤Decrease the level of hs-CRP (*p* < 0.05)	Wang et al. (2009)
31	People’s Hospital of Hainan Provincial	Liqi Huoxue dropping pills (LHDP)	*Litsea Lancilimba* Merr (Daguomujiangzi), *Conioselinum anthriscoides “Chuanxiong”* (Chuanxiong), *Allium chinense* G.Don (Xiebai)	8 weeks	(RCT) LHDP + CT *vs*. CT	102 (51/51)	①Angina pectoris attack times: I 1.83 ± 0.51 *vs*. C 3.20 ± 0.61 (times/week), (*p* < 0.05) ⑪Improve Chinese medical syndrome	③Improve the level of NO and reduce the level of ET-1 (*p* < 0.05) ⑤Decrease the levels of hs-CRP, PTX-3 and Lp-PLA2 (*p* < 0.05)	Li. (2019)
32	Heilongjiang Provincial Hospital of Traditional Chinese Medicine	Maixuekang capsule (MXK)	*Hirudo* (Shuizhi)	28 days	(RCT) MXK + CT *vs*. CT	60 (30/30)	②I 80.0% *vs*. C 53.3% (*p* < 0.05) ⑬I 93.3% *vs*. C 70.0% (*p* < 0.05)	⑨Decrease the level of whole blood viscosity (*p* < 0.05)	Li et al. (2017)
33	The Provincial People’s Hospital Affiliated to Fujian University of Traditional Chinese Medicine	Naoxintong capsule (NXT)	*Astragalus mongholicus* Bunge (Huangqi), *Hirudo* (Shuizhi), *Pheretima* (Dilong), *Scorpio* (Quanxie), *Angelica sinensis* (Oliv.) Diels (Danggui), *Conioselinum anthriscoides “Chuanxiong”* (Chuanxiong), *Salvia miltiorrhiza* Bunge (Danshen), *Paeonia anomala* subsp. *veitchii (*Lynch) D.Y.Hong & K.Y.Pan (Chishao), *Boswellia sacra* Flück. (Ruxiang), *Juglans regia* L*.* (Taoren), *Carthamus tinctorius* L*.* (Honghua), *Cinnamomum cassia* (L.) J. Presl (Guizhi), *Morus alba L.* (Sangzhi), *Achyranthes bidentata* Blume (Niuxi), *Commiphora myrrha* (T.Nees) Engl. (Moyao), *Spatholobus suberectus* Dunn (Jixueteng)	2 weeks	(RCT) NXT + CT *vs*. CT	62 (31/31)	① I 93.5% *vs*. C 71.0% (*p >* 0.05) Angina pectoris attack times: I 0.8 ± 0.4 *vs*. C 1.2 ± 0.5 (*p* < 0.05); angina pectoris duration: I 10.2 ± 1.8 *vs*. C 15.4 ± 1.4 (*p* < 0.05) ⑦ I 0.5 ± 0.1 *vs*. C 0.8 ± 0.3 (*p* < 0.05)	Unreported	Cheng. (2011)
34	Luan Group General Hospital	Naoxintong capsule (NXT)	—	3 months	(RCT) NXT + CT *vs*. CT	40 (20/20)	① I 75.0% *vs*. C 50.0% (*p* < 0.05)	⑩Improve coronary blood flow (*p* < 0.05)	Wei et al. (2010)
35	Jining First People’s Hospital	Pushen capsule (PSC)	*Reynoutria* multiflora (Thunb.) *Moldenke* (Heshouwu), *Typha angustifolia* L*.* (Puhuang), *Salvia miltiorrhiza* Bunge (Danshen), *Conioselinum anthriscoides “Chuanxiong”* (Chuanxiong), *Paeonia anomala* subsp*. veitchii* (Lynch) D.Y.Hong & K.Y.Pan (Chishao), *Alisma plantago-aquatica* subsp*. orientale* (Sam.) Sam. (Zexie), *Codonopsis pilosula* (Franch.) Nannf*.* (Dangshen), *Crataegus pinnatifida* Bunge (Shanzha)	12 weeks	(RCT) PSC + CT *vs*. CT	64 (32/32)	①I 90.62% *vs*. C 68.75% (*p* < 0.05)	⑤Decrease the level of CRP (*p* < 0.05) ⑧Reduce the levels of TG, TC, and LDL-C and increase the level of HDL-C (*p* < 0.05)	Cao et al. (2021)
36	Chinese Medicine Hospital Affiliated to Xinjiang Medical University	Qihong powder (QHP)	*Astragalus mongholicus* Bunge (Huangqi), *Cinnamomum cassia* (L.) J.Presl (Guizhi), *Rhodiola rosea* L*.* (Hongjingtian), *Salvia miltiorrhiza* Bunge (Danshen), *Alisma plantago-aquatica* subsp*. orientale* (Sam.) Sam. (Zexie), *Lepidium apetalum* Willd*.* (Tinglizi)	6 months	(RCT) QHP + CT *vs*. CT	184 (92/92)	①Angina pectoris attack times: I 1.0 ± 0.2 *vs*. C 0.6 ± 0.2 (*p* < 0.05)	③Decrease the level of ET-1 (*p* < 0.05) ⑤Reduce the levels of IL-6 and hs-CRP (*p* < 0.05) ⑩Improve coronary blood flow (*p* < 0.05)	Jiang et al. (2021)
37	Cangzhou Central Hospital	Qili Qiangxin capsule (QQC)	*Astragalus mongholicus* Bunge (Huangqi), *Panax ginseng* C.A.Mey. (Renshen), *Raphanus raphanistrum* subsp*. sativus* (L.) *Domin* (Fuzi), *Salvia miltiorrhiza* Bunge (Danshen), *Lepidium apetalum* Willd*.* (Tinglizi), *Polygonatum odoratum* (Mill.) *Druce* (Yuzhu), *Alisma plantago-aquatica* subsp*.* orientale (Sam.) Sam*.* (Zexie), *Cinnamomum cassia* (L.) J. Presl (Guizhi), *Carthamus tinctorius* L. (Honghua), *Periploca sepium* Bunge (Xiangjiapi), *Citrus aurantium* L*.* (Chenpi)	6 months	(RCT) QQC + CT *vs*. CT	60 (40/40)	①Angina pectoris attack times: I 1.7 ± 0.8 *vs*. C 1.1 ± 0.7 (*p* < 0.05)	③Improve the level of NO and reduce the levels of ET-1, HA, HS, and syndecan 1 (*p* < 0.05) ⑤Decrease the levels of hs-CRP and TNF-α (*p* < 0.05) ⑩Improve coronary blood flow (*p* < 0.05)	Zhang et al. (2021)
38	Hospital of Zhongshan, Affiliated to Guangzhou University of Chinese Medicine	Qingling decoction (QLD)	*Ilex pubescens* Hook. & Arn. (Maodongqing), *Zanthoxylum nitidum* (Roxb.) DC. (Liangmianzhen), *Scutellaria baicalensis* Georgi (Huangqin), *Hansenia forbesii* (H.Boissieu) Pimenov & Kljuykov (Qianghuo), *Saposhnikovia divaricata* (Turcz. ex Ledeb.) Schischk. (Fangfeng), *Clematis chinensis* Osbeck (Weilingxian), *Pinellia ternata* (Thunb.) Makino (Banxia), *Citrus × aurantium* L. (Juhong)	4 weeks	(RCT) QLD + CT *vs*. CT	30 (15/15)	①Angina pectoris attack times: I 3.15 ± 2.85 vs. C 11.37 ± 4.11 (*p* < 0.05); ⑪Improve Chinese medical syndrome (*p* < 0.05)	③Reduce the level of ET-1 (*p* < 0.05) ⑤Decrease the level of CRP (*p* < 0.05)	Lin et al. (2011)
39	Beijing Hospital of Integrated Traditional Chinese and Western Medicine	Qishen capsule (QS)	*Astragalus mongholicus* Bunge (Huangqi), *Salvia miltiorrhiza* Bunge (Danshen), *Panax ginseng* C.A.Mey. (Renshen), *Poria cocos* (Schw.) *Wolf* (Fuling), *Panax notoginseng* (Burkill) F.H.Chen (Sanqi), *Hirudo* (Shuizhi), *Carthamus tinctorius* L*.* (Honghua), *Conioselinum anthriscoides “Chuanxiong”* (Chuanxiong), *Crataegus pinnatifida* Bunge (Shanzha), *Typha angustifolia* L. (Puhuang), *Reynoutria multiflora* (Thunb.) *Moldenke* (Zhiheshouwu), *Pueraria montana* var. *lobata* (Willd.) Maesen & S.M.Almeida ex Sanjappa & *Predeep* (Gegen), *Scutellaria baicalensis Georgi* (Huangqin), *Scrophularia ningpoensis* Hemsl. (Xuanshen), *Glycyrrhiza uralensis* Fisch. ex DC*.* (Gancao)	12 weeks	(RCT) QS + CT *vs*. CT	120 (60/60)	①Decrease the frequency of angina pectoris attacks (*p* < 0.05) ④Improve the exercise tolerance (*p* < 0.05) ⑦Reduce the dosage of nitroglycerin required (*p* < 0.05)	⑤Reduce the levels of hs-CRP and SOD (*p* < 0.05)	Zhang et al. (2013b)
40	The Central Hospital of Jiamusi City	Qishen capsule (QS)	—	2 months	(RCT) QS + CT *vs*. CT	68 (34/34)	① I 88.24% *vs*. C 67.65% (*p* < 0.05) ⑦Reduce the dosage of nitroglycerin required (*p* < 0.05)	③Decrease the level of ET-1 and increase the level of NO (*p* < 0.05)	Shen. (2021)
41	The First Hospital of Jilin University	Qishen Yiqi dropping pills (QSYQ)	*Astragalus mongholicus* Bunge (Huangqi), *Salvia miltiorrhiza* Bunge (Danshen), *Panax notoginseng* (Burkill) F.H.Chen (Sanqi), *Dalbergia* odorifera T.C.Chen (Jiangxiang)	3 months	(RCT) QSYQ + CT *vs*. CT	60 (30/30)	① I 83.3% *vs*. C 33.3% (*p* < 0.05)	⑩ I 76.9% *vs*. C 43.5% (*p* < 0.05)	Kang et al. (2021)
42	Affiliated Hospital of Inner Mongolia Medical University	Safflower yellow pigment injection (SYPI)	*Carthamus tinctorius L.* (Honghua)	14 days	(RCT) SYPI + CT *vs*. CT	68 (36/32)	⑬I 75.0% *vs*. C 46.9% (*p* < 0.05)	⑤Decrease the level of hs-CRP (*p* < 0.05)	Wang and Wang. (2013)
43	The Second People’s Hospital of Fengrun District, Tangshan	*Salvia miltiorrhiza* and ligustrazine hydrochloride injection (SMLH)	*Salvia miltiorrhiza Bunge* (Danshen), *Conioselinum anthriscoides “Chuanxiong”* (Chuanxiong)	2 weeks	(RCT) SMLH + CT *vs*. CT	104 (52/52)	① I 96.15% *vs*. C 51.92% (*p* < 0.05)	Unreported	Yang and Zhang. (2013)
44	Affiliated Hospital of Traditional Chinese Medicine Research Institute in Heilongjiang Province	Shenwu Guanxin granules (SGG)	*Panax ginseng* C.A.Mey. (Hongshen), *Astragalus mongholicus* Bunge (Huangqi), *Lepidium apetalum* Willd. (Tinglizi), *Raphanus raphanistrum* subsp*. sativus (L.) Domin* (Fuzi), *Poria cocos* (Schw.) Wolf (Fuling), *Paeonia anomala* subsp*. veitchii* (Lynch) D.Y.Hong & K.Y.Pan (Chishao), *Lindera aggregata* (Sims) Kosterm. (Wuyao)	2 months	(RCT) SGG + CT *vs*. CT	64 (34/30)	①Angina pectoris attack times: I 4.14 ± 0.22 *vs*. C 9.58 ± 0.43 (*p* < 0.05); ④the total exercise time: I 459 ± 72 *vs*. C 421 ± 66 (*p* < 0.05); the time when the ST segment was depressed by 1 mV I 319 ± 65 *vs*. C 275 ± 61 (*p* < 0.05); the ST segment depression I 1.2 ± 0.4 *vs*. C 1.6 ± 0.5 (*p* < 0.05)	Unreported	Sun et al. (2007)
45	Department of Cardiology, Seventh Medical Center of Chinese PLA General Hospital	Shexiang Tongxin dripping pills (STP)	*Borneolum Syntheticum* (Bingpian), *Pulvis Fellis Ursi* (Xiongdanfen), *Calculus Bovis* (Niuhuang), *Salvia miltiorrhiza* Bunge (Danshen), *Venenum Bufonis* (Chansu), *Panax ginseng* C.A.Mey. (Renshen), *Moschus* (Shexiang)	3 months	(RCT) STP + CT *vs*. CT	106 (54/52)	⑬I 96.29% *vs*. C 78.84% (*p* < 0.05)	④The total exercise time: I 542 ± 87 *vs*. C 473 ± 76 (*p* < 0.05); the maximum amplitude of ST-segment depression I 0.5 ± 0.1 *vs*. C 0.8 ± 0.2 (*p* < 0.05); ⑫I 96.29% *vs*. C 75.00% *p* < 0.05	Gong et al. (2021)
46	Department of Pharmacy, Aviation Hospital	Shuxuening injection (SXN)	*Ginkgo biloba* L*.* (Yinxingye)	15 days	(RCT) SXN + CT *vs*. CT	50 (26/24)	①Reduced the frequency of angina pectoris attacks (*p* < 0.05)	①Decrease the frequency of typical angina occurrence (*p* < 0.05) ④Improve the result of the treadmill exercise test (*p* < 0.05)	Wu et al. (2010)
47	Longhua Hospital, Shanghai University of Traditional Chinese Medicine	Shuxuetong injection (SXT)	*Pheretima* (Dilong), *Hirudo* (Shuizhi)	7 days	(RCT) SXT + CT *vs*. CT	64 (32/32)	① I 93.75% *vs*. C 90.00% (*p* < 0.05) ② I 93.75% *vs*. C 73.33% (*p* < 0.05)	⑤Reduce the levels of hs-CRP, ICAM-1, and TNF-α (*p* < 0.05)	Mao et al. (2021)
48	Capital Medical University affiliated Beijing Anzhen Hospital	Sodium tanshinone II A sulfonate injection (SSI)	*Salvia miltiorrhiza* Bunge (Danshen)	14 days	(RCT) SSI + CT *vs*. CT	70 (35/35)	① I 94.2% *vs*. C 80.0% (*p* < 0.05)	③Reduce the level of ET-1 (*p* < 0.05) ⑤Decrease the levels of CRP and Fib (*p* < 0.05)	Zu et al. (2012)
49	Zhoukou Hospital of traditional Chinese Medicine	Tongluo Ningxin recipe (TNR)	*Astragalus mongholicus* Bunge (Huangqi), *Salvia miltiorrhiza* Bunge (Danshen), *Conioselinum anthriscoides “Chuanxion”* (Chuanxiong), *Angelica sinensis* (Oliv.) Diels (Danggui), *Paeonia anomala* subsp*. veitchii* (Lynch) D.Y.Hong & K.Y.Pan (Chishao), *Citrus aurantium* L. (Zhishi), *Carthamus tinctorius* L*.* (Honghua), *Hirudo* (Shuizhi)	3 months	(RCT) TNR + CT *vs*. CT	70 (35/35)	⑬I 94.29% *vs*. C 77.14% (*p* < 0.05)	⑤Decrease the levels of hs-CRP and IL-6 (*p* < 0.05) ⑩Improve coronary blood flow (*p* < 0.05)	Li. (2018)
50	The Second Affiliated People’s Hospital of Fujian University of Traditional Chinese Medicine	Tongmai Zhuyu decoction (TMZY)	*Panax notoginseng (*Burkill) F.H.Chen (Sanqi), *Cyathula officinalis* K.C.Kuan (Chuanniuxi), *Angelica sinensis* (Oliv.) Diels (Danggui), *Conioselinum anthriscoides “Chuanxiong”* (Chuanxiong), *Salvia miltiorrhiza* Bunge (Danshen), *Curcuma aromatica* Salisb*.* (Yujin), *Panax ginseng* C.A.Mey. (Renshen), *Astragalus mongholicus* Bunge (Huangqi), *Bupleurum chinense* DC. (Chaihu), *Citrus aurantium* L*.* (Zhiqiao), *Ilex pubescens* Hook. & Arn. (Maodongqing), *Glycyrrhiza* uralensis Fisch. ex DC. (Zhigancao)	4 weeks	(RCT) TMZY + CT *vs*. CT	40 (20/20)	① I 85% *vs*. C 50% (*p* < 0.05)	Unreported	Su and Luo. (2014)
51	Shuguang Hospital Affiliated to Shanghai University of Traditional Chinese Medicine	Wenyang Huoxue recipe (WHR)	*Raphanus raphanistrum* subsp*. sativus (L.)* Domin (Fuzi), *Angelica sinensis* (Oliv.) Diels (Danggui), *Typha angustifolia* L*.* (Puhuang), *Citrus aurantium* L*.* (Zhiqiao), *Platycodon grandiflorus* (Jacq.) A.DC. (Jiegeng), *Paeonia anomala* subsp*. veitchii* (Lynch) D.Y.Hong & K.Y.Pan (Chishao), *Paeonia lactiflora* Pall*.* (Baishao), *Glycyrrhiza uralensis* Fisch. ex DC. (Gancao)	1 month	(RCT) WHR + CT *vs*. CT	60 (30/30)	①Angina pectoris attack times: I 0.83 ± 0.75 *vs*. C 1.77 ± 0.70 (*p* < 0.05); angina pectoris duration: I 1.03 ± 0.42 vs. C 0.87 ± 0.43(*p* < 0.05) ⑦Reduce the dosage of nitroglycerin required (*p* < 0.05) ⑬I 96.7% *vs*. C 86.9% (*p* < 0.05)	③Improve the level of NO and reduce the level of ET-1 (*p* < 0.05) ⑧Decrease the levels of TG, TC, and LDL-C and increase the level of HDL-C (*p* < 0.05)	Xue et al. (2014)
52	East City Hospital Affiliated to the University of Shanghai for Science and Technology	Wide chest aerosol (WCA)	*Santalum album* L*.* (Tanxiang), *Piper longum* L. (Bibo), *Asarum sieboldii* Miq*.* (Xixin), *Alpinia* galanga (L*.) Willd.* (Gaoliangjiang), *Borneolum Syntheticum* (Bingpian)	1 month	(RCT) WCA + CT *vs.* CT	72 (36/36)	① I 91.7% *vs*. C 72.2% (*p* < 0.05)	③Increase the level of NO and decrease the level of ET-1 (*p* < 0.05)	Liu et al. (2021b)
53	the Second Affiliated Hospital of Shantou University Medical College	Wuling capsule (WLC)	*Xylaria Nigripes* (Wulingjunfen)	12 weeks	(RCT) WLC + CT *vs*. CT	62 (31/31)	①Angina pectoris attack times: I 6.2 ± 1.8 vs. C 9.3 ± 2.9 (*p* < 0.05)	Unreported	Zhou. (2021)
54	Liaocheng Second People’s Hospital Affiliated to Shandong Medical University	Xinkeshu tablets (XKS)	*Crataegus pinnatifida* Bunge (Shanzha), *Salvia miltiorrhiza* Bunge (Danshen), *Pueraria montana* var. *lobata* (Willd.) Maesen & S.M.Almeida ex Sanjappa & *Predeep* (Gegen), *Panax notoginseng* (Burkill) F.H.Chen (Sanqi), *Dolomiaea costus* (Falc.) Kasana & A.K.Pandey (Muxiang)	6 months	(RCT) XKS + CT *vs*. CT	90 (45/45)	① I 84.44% *vs*. C 63.33% (*p* < 0.05)	③Improve the level of NO and reduce the level of ET-1 (*p* < 0.05); improve FMD (*p* < 0.05)	Feng et al. (2017)
55	The Second Hospital of Shijiazhuang	Xinkeshu tablets (XKS)	*Crataegus pinnatifida* Bunge (Shanzha), *Salvia miltiorrhiza* Bunge (Danshen), *Pueraria montana* var. *lobata* (Willd.) Maesen & S.M.Almeida ex Sanjappa & Predeep (Gegen), *Panax notoginseng* (Burkill) F.H.Chen (Sanqi), *Dolomiaea costus* (Falc.) Kasana & A.K.Pandey (Muxiang)	3 months	(RCT) XKS + CT *vs*. CT	60 (30/30)	① I 90% *vs*. C 67% (*p* < 0.05)	③Increase the level of NO (*p* < 0.05); ⑤Decrease the level of hs-CRP (*p* < 0.05)	Jia et al. (2019b)
56	First Hospital of Shijiazhuang	Xinnaoning capsule (XNN)	*Ginkgo biloba* L. (Yinxingye), *Buxus Microphylla* (Xiaoyehuangyang), *Salvia miltiorrhiza* Bunge (Danshen), *Litsea Lancilimba* Merr. (Daguomujiangzi), *Allium chinense* G.Don (Xiebai)	3 months	(RCT) XNN + CT *vs.* CT	48 (25/23)	①Angina pectoris attack times: I 5.3 ± 1.7 *vs*. C 8.9 ± 2.6 (*p* < 0.05)	Unreported	Ren et al. (2018)
57	Zhengzhou Central Hospital Affiliated to Zhengzhou University	Xuefu Zhuyu decoction (XFZY)	*Juglans regia* L*.* (Taoren), *Cornus officinalis* Siebold & Zucc. (Shanzhuyu), *Paeonia anomala* subsp*. veitchii* (Lynch) D.Y.Hong & K.Y.Pan (Chishao), *Reynoutria multiflora* (Thunb.) *Moldenke* (Heshouwu), *Carthamus tinctorius* L. (Honghua), *Angelica sinensis* (Oliv.) Diels (Danggui), *Rehmannia glutinosa* (Gaertn.) DC. (Dihuang)	4 weeks	(RCT) XFZY + CT *vs*. CT	102 (51/51)	① I 94.2% *vs*. C 78.4% (*p* < 0.05)	③Increase the level of NO and decrease the level of ET-1 (*p* < 0.05) ⑤Decrease the levels of hs-CRP, IL-6, and TNF-α (*p* < 0.05) ⑧Reduce the levels of TG, TC, and LDL-C and increase the level of HDL-C (*p* < 0.05) ⑨Reduce the levels of whole blood viscosity, plasma specific viscosity, and platelet adhesion rate (*p* < 0.05)	Zhu. (2019)
58	The Second Affiliated Hospital of Shandong University of Traditional Chinese Medicine	Yinxing Mihuan Oral Liquid (YXMH)	*Ginkgo biloba* L*.* (Yinxingye), *Gastrodia elata* Blume (Tianma)	2 weeks	(RCT) YXMH + CT *vs*. CT	84 (42/42)	① I 92.9% *vs*. C 78.6% (*p* < 0.05) angina pectoris attack times: I 1.7 ± 0.8 *vs*. C 3.8 ± 1.6 (*p* < 0.05); angina pectoris pain score: I 2.9 ± 1.1 *vs*. C 5.1 ± 1.7 (*p* < 0.05); angina pectoris duration: I 1.7 ± 0.6 *vs*. C 3.5 ± 1.2 (*p* < 0.05)	③Increase the level of NO and reduce the levels of ET-1 and AngⅡ (*p* < 0.05) ⑤Decrease the level of hs-CRP (*p* < 0.05)	Xuan et al. (2020)
59	Hebei General Hospital for Retired Soldiers	Yiqi Fumai Lyophilized injection (YQFM)	*Panax ginseng* C.A.Mey. (Hongshen), *Ophiopogon japonicus* (Thunb.) Ker Gawl. (Maidong), *Schisandra chinensis* (Turcz.) Baill. (Wuweizi)	14 days	(RCT) YQFM + CT *vs*. CT	80 (40/40)	① I 87.5% *vs*. C 65.0% (*p* < 0.05)	Unreported	Guo et al. (2020)
60	Jilin Academy of Traditional Chinese Medicine	Yiqi Huoxue Huatan formula granules (YHH)	*Trichosanthes kirilowii* Maxim*.* (Gualou), *Allium chinense* G.Don (Xiebai), *Pinellia ternata* (Thunb.) *Makino* (Qingbanxia), *Astragalus mongholicus* Bunge (Huangqi), *Salvia miltiorrhiza* Bunge (Danshen), *Panax notoginseng* (Burkill) F.H.Chen (Sanqi), *Rhodiola rosea* L*.* (Hongjingtian)	4 weeks	(RCT) YHH + CT *vs*. CT	68 (34/34)	①Angina pectoris attack times: I 2.35 ± 0.92 *vs*. C 3.18 ± 1.22 (*p* < 0.05); angina pectoris pain level: I 2.61 ± 0.33 *vs*. C 3.50 ± 0.58 (*p* < 0.05); angina pectoris duration: I 2.65 ± 1.07 *vs*. C 3.47 ± 1.02 (*p* < 0.05); ④improve the treadmill exercise test	⑤Decrease the level of hs-CRP (*p* < 0.05)	Zhou. (2018)
61	Shuguang Hospital Affiliated to Shanghai University of Traditional Chinese Medicine	Yiqi Huoxue Huatan recipe (YHHR)	*Astragalus mongholicus* Bunge (Huangqi), *Salvia miltiorrhiza* Bunge (Danshen), *Trichosanthes kirilowii* Maxim*.* (Gualou), *Hirudo* (Shuizhi), *Coptis chinensis* Franch*.* (Huanglian)	3 months	(RCT) YHHR + CT *vs*. CT	44 (22/22)	①Angina pectoris attack times: I 0.84 ± 0.27 *vs*. C 1.98 ± 0.77 (*p* < 0.05) ⑦Reduce the dosage of nitroglycerin required (*p* < 0.05) ⑪Improve Chinese medical syndrome (*p* < 0.05)	⑩Improved coronary blood flow (*p* < 0.05)	Huang et al. (2019)
62	Shanghai Municipal Hospital of Traditional Chinese Medicine Affiliated to Shanghai University of Traditional Chinese Medicine	Yiqi Huoxue recipe (YHR)	*Astragalus mongholicus* Bunge (Huangqi), *Scorpio* (Quanxie), *Cyperus brevifolius* (Rottb.) Hassk. (Wugong), *Juglans regia* L*.* (Taoren), *Citrus aurantium* L*.* (Zhiqiao), *Salvia miltiorrhiza* Bunge (Danshen)	2 months	(RCT) YHR + CT *vs*. CT	61 (31/30)	②I 87.10% *vs*. C 73.33% (*p* < 0.05) ⑬I 90.32% *vs*. C 76.67% (*p* < 0.05)	⑨EF: I 68.73 ± 4.75 *vs*. C 58.96 ± 3.88 *p* > 0.05	Feng et al. (2013)
63	Linyi Hospital of Traditional Chinese Medicine	Yiqi Huoxue (YQHX)	*Astragalus mongholicus* Bunge (Huangqi), *Ophiopogon japonicus* (Thunb.) Ker Gawl. (Maidong), *Schisandra chinensis* (Turcz.) Baill. (Wuweizi), *Cinnamomum cassia* (L.) J.Presl (Guizhi), *Rehmannia glutinosa* (Gaertn.) DC. (Dihuang), *Paeonia lactiflora* Pall. (Baishao), *Conioselinum anthriscoides “Chuanxiong”* (Chuanxiong), *Salvia miltiorrhiza* Bunge (Danshen), *Borneolum Syntheticum* (Bingpian), *Hirudo* (Shuizhi), *Corydalis yanhusuo* (Y.H.Chou & Chun C.Hsu) W.T.Wang ex Z.Y.Su & C.Y.Wu (Yanhusuo), *Poria cocos* (Schw.) Wolf (Fuling), *Citrus aurantium* L*.* (Chenpi), *Glycyrrhiza uralensis* Fisch. ex DC. (Zhigancao)	2 weeks	(RCT) YQHX + CT *vs*. CT	80 (40/40)	① I 92.5% *vs*. C 75% (*p* < 0.05)	③Reduce the level of ET-1 (*p* < 0.05) ⑤Decrease the level of hs-CRP (*p* < 0.05)	Tian et al. (2015)
64	Shanghai Municipal Hospital of Traditional Chinese Medicine Affiliated to Shanghai University of Traditional Chinese Medicine	Yiqi Tongluo recipe (YTR)	*Astragalus mongholicus* Bunge (Huangqi), *Scorpio* (Quanxie), *Angelica sinensis* (Oliv.) Diels (Danggui), *Hirudo* (Shuizhi), *Salvia miltiorrhiza* Bunge (Danshen), *Carthamus tinctorius* L. (Honghua), *Paeonia anomala* subsp*. veitchii* (Lynch) D.Y.Hong & K.Y.Pan (Chishao), *Conioselinum anthriscoides “Chuanxiong”* (Chuanxiong), *Citrus aurantium* L*.* (Zhishi)	3 months	(RCT) YTR + CT *vs*. CT	59 (30/29)	① I 93.33% *vs*. C 75.86% (*p* < 0.05) ② I 90.00% *vs*. C 72.41% (*p* < 0.05)	③Improve FMD (*p* < 0.05) ⑤Decrease the level of hs-CRP (*p* < 0.05) ⑩Improve coronary blood flow (*p* < 0.05)	Wu et al. (2016)
65	Linhai Hospital of Traditional Chinese Medicine	Yishen Jieyu Tongmai decoction (YJT)	*Rehmannia glutinosa* (Gaertn.) DC. (Shudihuang), *Cornus officinalis* Siebold & Zucc. (Shanzhuyu), *Ophiopogon japonicus* (Thunb.) Ker Gawl. (Maidong), *Lycium barbarum* L*.* (Gouqizi), *Bupleurum chinense* DC. (Chaihu), *Curcuma aromatica* Salisb*.* (Yujin), *Astragalus mongholicus* Bunge (Huangqi), *Angelica sinensis* (Oliv.) Diels (Danggui), *Pheretima* (Dilong), *Glycyrrhiza uralensis* Fisch. ex DC. (Gancao)	4 weeks	(RCT) YJT + CT *vs*. CT	106 (53/53)	⑬I 90.5% *vs*. C 79.2% (*p* < 0.05)	⑤Decrease the level of CRP (*p* < 0.05)	Chen and Song. (2019)
66	Tai’an Hospital of Traditional Chinese Medicine	Yixin Tongluo capsule (YTC)	*Astragalus mongholicus* Bunge (Huangqi), *Panax ginseng* C.A.Mey. (Renshen), *Ophiopogon japonicus* (Thunb.) Ker Gawl. (Maidong), *Salvia miltiorrhiza* Bunge (Danshen), *Dalbergia odorifera* T.C.Chen (Jiangxiang), *Citrus aurantium* L*.* (Zhiqiao), *Conioselinum anthriscoides “Chuanxiong”* (Chuanxiong), *Poria cocos* (Schw.) Wolf (Fuling), *Pinellia ternata* (Thunb.) Makino (Banxia), *Trichosanthes kirilowii* Maxim. (Gualou), *Allium chinense* G.Don (Xiebai), *Citrus aurantium* L*.* (Chenpi), *Glycyrrhiza uralensis* Fisch. ex DC. (Gancao)	8 weeks	(RCT) YTC + CT *vs*. CT	120 (60/60)	⑪I 91.67% *vs*. C 68.33% (*p* < 0.05)	⑩Coronary blood flow significantly improved ⑭LVEF: I 63.07 ± 5.28 *vs*. C 53.17 ± 4.36 ⑥I 18.29 ± 3.16 *vs*. C 23.67 ± 3.21, *p* < 0.05	Meng. (2018)
67	Liuzhou Hospital of Traditional Chinese Medicine	Yixin yin (YXY)	*Astragalus mongholicus* Bunge (Huangqi), *Codonopsis pilosula* (Franch.) Nannf*.* (Dangshen), *Salvia miltiorrhiza* Bunge (Danshen), *Dalbergia odorifera* T.C.Chen (Jiangxiang), *Paeonia anomala* subsp*. veitchii* (Lynch) D.Y.Hong & K.Y.Pan (Chishao), *Conioselinum anthriscoides “Chuanxiong”* (Chuanxiong), *Schisandra chinensis* (Turcz.) Baill*.* (Wuweizi), *Ophiopogon japonicus* (Thunb.) Ker Gawl. (Maidong), *Trichosanthes kirilowii* Maxim. (Gualoupi), *Pueraria montana* var. *lobata* (Willd.) Maesen & S.M.Almeida ex Sanjappa & *Predeep* (Gegen), *Allium chinense* G.Don (Xiebai), *Cinnamomum cassia* (L.) J. Presl (Guizhi), *Glycyrrhiza uralensis* Fisch. ex DC*.* (Zhigancao)	6 months	(RCT) YXY + CT *vs*. CT	60 (30/30)	①I 80.0% *vs*. C 33.3% (*p* < 0.05) ④the total exercise time: I 10.18 ± 3.35 *vs*. C 8.45 ± 2.51 (*p* < 0.05)	⑩Improve coronary blood flow (*p* < 0.05)	Long et al. (2010)
68	Beijing Anzhen Hospital, Capital Medical University	Yixinshu capsule (YXS)	*Panax ginseng* C.A.Mey*.* (Renshen), *Salvia miltiorrhiza* Bunge (Danshen), *Ophiopogon japonicus* (Thunb.) Ker Gawl. (Maidong), *Astragalus mongholicus* Bunge (Huangqi), *Conioselinum anthriscoides “Chuanxiong”* (Chuanxiong), *Schisandra chinensis* (Turcz.) Baill. (Wuweizi), *Crataegus pinnatifida* Bunge (Shanzha)	24 weeks	(RCT) YXS + CT *vs*. CT	366 (200/166)	① I 89.50% *vs*. C 82.53% (*p* > 0.05); angina pectoris attack times: I 2.33 ± 1.26 *vs*. C 4.33 ± 2.13 (*p* < 0.05);	③Improve FMD (*p* < 0.05)	Zhu and Chen. (2014)
							angina pectoris duration: I 1.64 ± 1.10 *vs*. C 2.79 ± 1.48 (*p* < 0.05); ② I 65.00% *vs*. C 54.46% (*p* > 0.05)		
69	The Third People’s Hospital of Dalian	Yuxintong capsule (YXT)	*Corydalis yanhusuo* (Y.H.Chou & Chun C.Hsu) W.T.Wang ex Z.Y.Su & C.Y.Wu (Yanhusuo), *Panax ginseng* C.A.Mey*.* (Hongshen), *Panax notoginseng* (Burkill) F.H.Che*n* (Sanqi)	4 weeks	(RCT) YXT + CT *vs.* CT	82 (41/41)	① I 97.56% *vs*. C 80.49% (*p* < 0.05) ④The total exercise time: I 8.94 ± 0.36 *vs*. C 6.47 ± 0.32 (*p* < 0.05); the time when the ST segment was depressed by 1 mV I 7.43 ± 0.38 *vs*. C 5.96 ± 0.35 (*p* < 0.05); the ST segment depression I 0.51 ± 0.06 *vs*. C 0.92 ± 0.04 (*p* < 0.05)	③Increase the level of NO and reduce the level of ET-1 (*p* < 0.05); ⑤Decrease the levels of CRP, IL-1, and IL-6 (*p* < 0.05)	Wu et al. (2018)
70	Shijiazhuang Hospital of Traditional Chinese Medicine	Zhima Xiatan Huoxue recipe (ZXH)	*Astragalus mongholicus* Bunge (Huangqi), *Citrus aurantium* L. (Chenpi), *Arisaema erubescens* (Wall.) Schott (Dannanxing), *Angelica sinensis* (Oliv.) Diels (Danggui), *Juglans regia* L. (Taoren), *Scorpio* (Quanxie), *Conioselinum anthriscoides “Chuanxiong”* (Chuanxiong), *Zaocys* (Wushaoshe), *Paeonia anomala* subsp*. veitchii* (Lynch) D.Y.Hong & K.Y.Pan (Chishao), *Spatholobus suberectus* Dunn (Jixueteng), *Carthamus tinctorius* L*.* (Honghua), *Salvia miltiorrhiza* Bunge (Danshen), *Morus alba* L*.* (Sangzhi), *Pinellia ternata (*Thunb.) Makino (Banxia)	1 month	(RCT) WLC + CT *vs*. CT	72 (36/36)	Unreported	⑤Decrease the levels of IL-2, IL-6, and hs-CRP (*p* < 0.05); ⑩Improve coronary blood flow (*p* < 0.05) ⑨Improve EF (*p* < 0.05) ③Reduce the levels of HA, HS, and syndecan 1 (*p* < 0.05)	Liu et al. (2021a)
71	The First Affiliated Hospital of Heilongjiang University of Chinese Medicine	Zhishi Xiebai Guizhi decoction (ZXB)	*Citrus aurantium* L*.* (Zhishi), *Allium chinense* G.Don (Xiebai), *Cinnamomum cassia* (L.) J. Presl (Guizhi), *Trichosanthes kirilowii* Maxim*.* (Gualou), *Magnolia officinalis* Rehder & E.H.Wilson (Houpo)	2 weeks	(RCT) ZXB + CT *vs*. CT	72 (36/36)	① I 94.4% *vs*. C 75.0% (*p* < 0.05) ② I 88.9% *vs*. C 66.7% (*p* < 0.05)	Unreported	Shi and Cui. (2019)

①Angina symptom improvement rate. ②Electrocardiograph improvement rate. ③Vascular endothelial function. ④Treadmill exercise test. ⑤The level of inflammatory indexes. ⑥Index of microvascular resistance. ⑦The dosage of nitroglycerin. ⑧Blood lipid parameters. ⑨Hemodynamic indexes. ⑩Coronary microcirculation function. ⑪ The effective rate of Chinese medical syndrome. ⑫Coronary flow reserve. ⑬Clinical efficacy. ⑭Cardiac function.

**TABLE 2:** Clinical evidence of TCM for typical manifestations of CMVD.

Clinical evidence	TCM
Clinical manifestations	
Angina pectoris	Tongxinluo capsule, Shexiang Baoxin pill, Qishen capsule, Qishen Yiqi dropping pills, Yinxing Mihuan oral solution, Yixinshu capsule, Xinkeshu tablets, Danlou tablets, Yindan Xinnaotong soft capsule, Yuxintong capsule, Wide chest aerosol, Le Mai pill, Naoxintong capsule, Liqi Huoxue dropping pills, Ginkgo Biloba capsule, Maixuekang capsule, Wuling capsule, Xinnaoning capsule, Pushen capsule, Qili Qiangxin capsule, compound Danshen dripping pills, Guanxin Danshen drop pills, Guanxin Shutong capsule, Naoxintong capsule, Shexiang Tongxin dripping pills, Danhong injection, Shuxuening injection, Yiqi Fumai Lyophilized injection, *Salvia miltiorrhiza* and ligustrazine hydrochloride injection, Ginkgo Damo injection, ligustrazine injection, safflower yellow pigment injection, sodium tanshinone ⅡA sulfonate injection, Liqi Huatan Huoxue formula, Huayu Fuyuan capsule, Huoxue Zhuyu decoction, Fufang Wenban decoction, Shenwu Guanxin granules, Bushen Huoxue granules, Tongmai Zhuyu decoction, Yiqi Huoxue, Yiqi Huoxue Huatan formula granules, Qingling decoction, Yishen Jieyu Tongmai decoction, Yixin yin, Yiqi Tongluo recipe, Yiqi Huoxue Huatan recipe, Yiqi Huoxue recipe, Wenyang Huoxue decoction, Tongluo Ningxin recipe, Danshen decoction, Danggui Sini decoction, Erxian decoction, Zhishi Xiebai Guizhi decoction, Xuefu Zhuyu decoction
Anxiety and depression	Xinkeshu tablets, Wuling capsule, Xinnaoning capsule
ECG	Tongxinluo capsule, Danlou tablets, Le Mai pill, Ginkgo Biloba capsule, Maixuekang capsule, Danhong injection, Shuxuening injection, Shuxuetong injection, Ginkgo Damo injection, Huoxue Tongmai Yixin decoction, Bushen Huoxue granules, Tongmai Zhuyu decoction, Yiqi Huoxue Huatan formula granules, Qingling decoction, Yiqi Tongluo recipe, Yiqi Huoxue recipe, Tongluo Ningxin recipe, Danshen decoction, Zhishi Xiebai Guizhi decoction
TCM syndrome	Qishen capsule, Xinkeshu tablets, Liqi Huoxue dropping pills, compound Danshen dripping pills, Yixin Tongluo capsule, Shuxuetong injection, Yiqi Fumai Lyophilized injection, Liqi Huatan Huoxue formula, Huoxue Tongmai Yixin decoction, Huoxue Zhuyu decoction, Yiqi Huoxue Huatan recipe, Wenyang Huoxue decoction, Danshen decoction, Danggui Sini decoction
The dosage of nitroglycerin	Shexiang Baoxin pill, Qishen capsule, Naoxintong capsule, Yiqi Huoxue Huatan recipe, Wenyang Huoxue decoction
The total time of treadmill exercise	Tongxinluo capsule, Shexiang Baoxin pill, Qishen capsule, Yuxintong capsule, Shexiang Tongxin dripping pills, Danhong injection, Huayu Fuyuan capsule, Huoxue Tongmai Yixin decoction, Shenwu Guanxin granules, Yiqi Huoxue Huatan formula granules, Yixin yin, Danggui Sini decoction, Erxian decoction
The time of ST-segment depression of 1 mm	Tongxinluo capsule, Shexiang Baoxin pill, Yuxintong capsule, Danhong injection, Huoxue Tongmai Yixin decoction, Shenwu Guanxin granules, Danggui Sini decoction, Erxian decoction
The maximum amplitude of ST-segment depression	Tongxinluo capsule, Shexiang Baoxin pill, Yuxintong capsule, Shexiang Tongxin dripping pills, Huayu Fuyuan capsule, Huoxue Tongmai Yixin decoction, Danggui Sini decoction, Erxian decoction
Coronary microcirculation	
FMD	Yixinshu capsule, Xinkeshu tablets, Liqi Huoxue dropping pills, ligustrazine injection, compound *Salvia miltiorrhiza* injection, Yiqi Tongluo recipe
CFR	Qishen Yiqi dropping pills, Compound Danshen dripping pills, Shexiang Tongxin dripping pills, Huoxue Tongmai Yixin decoction, Qihong powder
IMR	Yindan Xinnaotong soft capsule Yixin Tongluo capsule
TIMI	Compound Danshen dripping pills, Guanxin Danshen drop pills, Guanxin Shutong capsule, Naoxintong capsule, Ginkgo Damo injection, Yixin yin, Yiqi Tongluo recipe, Yiqi Huoxue Huatan recipe, Tongluo Ningxin recipe, Zhima Xiaotan Huoxue recipe, Danqi Tongmai capsule
Laboratory finding	
NO	Tongxinluo capsule, Shexiang Baoxin pill, Qishen capsule, Yinxing Mihuan oral solution, Xinkeshu tablets, Yindan Xinnaotong soft capsule, Yuxintong capsule, wide chest aerosol, Liqi Huoxue dropping pills, Qili Qiangxin capsule, Dengzhan Shenmai capsule, Shexiang Baoxin pill, compound *Salvia miltiorrhiza* injection, Liqi Huatan Huoxue formula, Huayu Fuyuan capsule, Huoxue Tongmai Yixin decoction, Huoxue Zhuyu decoction, Fufang Wenban decoction, Bushen Huoxue granules, Wenyang Huoxue decoction, Xuefu Zhuyu decoction
ET-1	Tongxinluo capsule, Shexiang Baoxin pill, Qishen capsule, Qishen Yiqi dropping pills, Yinxing Mihuan oral solution, Xinkeshu tablets, Yindan Xinnaotong soft capsule, Yuxintong capsule, wide chest aerosol, Liqi Huoxue dropping pills, Qili Qiangxin capsule, Dengzhan Shenmai capsule, Shexiang Baoxin pill, Danhong injection, compound *Salvia miltiorrhiza* injection, Liqi Huatan Huoxue formula, Huayu Fuyuan capsule, Huoxue Tongmai Yixin decoction, Huoxue Zhuyu decoction, Fufang Wenban decoction, Bushen Huoxue granules, Yiqi Huoxue, Qingling decoction, Qihong powder, Wenyang Huoxue decoction, Xuefu Zhuyu decoction, Danqi Tongmai capsule
CRP	Shexiang Baoxin pill, Yinxing Mihuan oral solution, Yindan Xinnaotong soft capsule, Yuxintong capsule, Liqi Huoxue dropping pills, Shuxuening injection, compound *Salvia miltiorrhiza* injection, Yiqi Huoxue, Qingling decoction, Yishen Jieyu Tongmai decoction
hs-CRP	Shexiang Baoxin pill, Xinkeshu tablets, Yindan Xinnaotong soft capsule, Pushen capsule, Qili Qiangxin capsule, compound Danshen dripping pills, Dengzhan Shenmai capsule, Shexiang Baoxin pill, Shuxuetong injection, safflower yellow pigment injection, sodium tanshinone ⅡA sulfonate injection, Danhong injection, Liqi Huatan Huoxue formula, Huayu Fuyuan capsule, Huoxue Tongmai Yixin decoction, Huoxue Zhuyu decoction, Fufang Wenban decoction, Yiqi Huoxue Huatan formula granules, Qihong powder, Yiqi Tongluo recipe, Tongluo Ningxin recipe, Xuefu Zhuyu decoction, Zhima Xiaotan Huoxue recipe
IL-1	Shexiang Baoxin pill, Yindan Xinnaotong soft capsule, Yuxintong capsule
IL-6	Shexiang Baoxin pill, Yindan Xinnaotong soft capsule, Yuxintong capsule, ligustrazine injection, compound *Salvia miltiorrhiza* injection, Qihong powder, Tongluo Ningxin recipe, Xuefu Zhuyu decoction, Zhima Xiaotan Huoxue recipe
TNF-α	Qili Qiangxin capsule, Shuxuetong injection, ligustrazine injection, compound *Salvia miltiorrhiza* injection, Xuefu Zhuyu decoction
Ang II	Yinxing Mihuan oral solution, Danqi Tongmai capsule
BNP	Ginkgo Biloba capsule, Qihong powder, Zhima Xiaotan Huoxue recipe
cTnI	Ginkgo Biloba capsule, compound Danshen dripping pills
MDA	Compound Danshen dripping pills, compound *Salvia miltiorrhiza* injection
Endothelial glycocalyx	Qili Qiangxin capsule, Zhima Xiaotan Huoxue recipe
Blood lipid	Shexiang Baoxin pill, Ginkgo Biloba capsule, Pushen capsule, Wenyang Huoxue decoction, Xuefu Zhuyu decoction
Hemorheology	Maixuekang capsule, compound *Salvia miltiorrhiza* injection
Cardiac function	Danlou tablets, Dengzhan Shenmai capsule, Yixin Tongluo capsule, Zhima Xiaotan Huoxue recipe, Danqi Tongmai capsule

#### 5.2.1 CPMs for CMVD treatment

Several clinical studies have shown that CPMs injections can play an important role in improving clinical symptoms, improving exercise tolerance, enhancing coronary microcirculation function, and regulating abnormal biomarkers and other aspects in CMVD patients.

1) Tongxinluo capsules (Liu et al., 2013; Wang, 2016; Fang et al., 2018), Shexiang Baoxin pills (Zhang W. N. et al., 2013; Wu et al., 2019), Qishen capsules (Zhang Y. L. et al., 2013; Shen, 2021), Qishen Yiqi dropping pills (Kang et al., 2021), Yinxing Mihuan oral solutions (Xuan et al., 2020), Yixinshu capsules (Zhu and Chen, 2014), Xinkeshu tablets (Feng et al., 2017; Jia W. M. et al., 2019), Danlou tablets (Zheng et al., 2019), Yindan Xinnaotong soft capsules (Wang et al., 2019; Wang and Li, 2022), Yuxintong capsules (Wu et al., 2018), Wide chest aerosols (Liu T. H. et al., 2021), Le Mai pills (Zhang and Xie, 2020), Naoxintong capsules (Cheng, 2011), Liqi Huoxue dropping pills (Li, 2019), Ginkgo Biloba capsules (Zou et al., 2016), Maixuekang capsules (Li et al., 2017), Wuling capsules (Zhou, 2021), Xinnaoning capsules (Ren et al., 2018), Pushen capsules (Cao et al., 2021), Qili Qiangxin capsules (Zhang et al., 2021), compound Danshen dripping pills (Zhang and Chen, 2020), Guanxin Danshen dropping pills (Fang et al., 2013), Guanxin Shutong capsules (Li, 2014), Dengzhan Shenmai capsules (Liu et al., 2017), Naoxintong capsules (Wei et al., 2010), and Shexiang Tongxin dropping pills (Gong et al., 2021) relieved angina symptoms and improved ECG results; Shexiang Baoxin pills (Zhang W. N. et al., 2013; Wu et al., 2019), Qishen capsules (Zhang Y. L. et al., 2013; Shen, 2021), and Naoxintong capsules (Cheng, 2011) decreased the nitroglycerin dosage needed; and Tongxinluo capsules (Fang et al., 2018), Shexiang Tongxin dropping pills (Gong et al., 2021), Qishen capsules (Zhang Y. L. et al., 2013), and Yuxintong capsules (Wu et al., 2018) improved exercise tolerance. 2) Shexiang Baoxin pills (Zhang, 2019), Qishen Yiqi dropping pills (Kang et al., 2021), compound Danshen dropping pills (Zhang and Chen, 2020), Guanxin Danshen dropping pills (Fang et al., 2013), Guanxin Shutong capsules (Li, 2014), and Shexiang Tongxin dropping pills (Gong et al., 2021) effectively improved CFRs; Yindan Xinnaotong soft capsules (Wang and Li, 2022) and Yixin Tongluo capsules (Meng, 2018) improved IMR values; and Guanxin Danshen dropping pills (Fang et al., 2013), Guanxin Shutong capsules (Li, 2014), compound Danshen dropping pills (Zhang and Chen, 2020), and Naoxintong capsules (Wei et al., 2010) improved TIMI blood flow grading. 3) Tongxinluo capsules (Wang, 2016), Shexiang Baoxin pills (Jin, 2018), Qishen capsules (Shen, 2021), Xinkeshu tablets (Feng et al., 2017; Jia W. M. et al., 2019), Yuxintong capsules (Wu et al., 2018), wide chest aerosols (Liu T. H. et al., 2021), Yindan Xinnaotong soft capsules (Wang and Li, 2022), Liqi Huoxue dropping pills (Li, 2019), and Qili Qiangxin capsules (Zhang et al., 2021) increased NO levels and decreased ET-1 levels; Yixinshu capsules (Zhu and Chen, 2014), Xinkeshu tablets (Feng et al., 2017), and Liqi Huoxue dropping pills (Li, 2019) effectively improved flow-mediated dilation (FMD); Yinxing Mihuan oral solutions (Xuan et al., 2020), Yindan Xinnaotong soft capsules (Wang et al., 2019; Wang and Li, 2022), and Yuxintong capsules (Wu et al., 2018) decreased the levels of hs-CRP, IL-6, IL-1, and tumor necrosis factor-α (TNF-α); Liqi Huoxue dropping pills (Li, 2019) decreased the levels of hs-CRP, Pentraxins 3 (PTX-3), and lipoprotein-associated phospholipase A2 (Lp-PLA2); Ginkgo Biloba capsules (Zou et al., 2016) decreased cardiac troponin I (cTnI) and BNP levels; Qili Qiangxin capsules (Zhang et al., 2021) decreased the levels of TNF-α, hs-CRP, hyaluronic acid, heparan sulphate, and syndecan 1; compound Danshen dropping pills (Zhang and Chen, 2020) decreased the levels of cTnI creatine kinase-MB (CK-MB) and malondialdehyde (MDA); and Shexiang Baoxin Pills (Zhang W. N. et al., 2013; Wu et al., 2019), Qishen capsules (Shen, 2021), Ginkgo Biloba capsules (Zou et al., 2016), and Pushen capsules (Cao et al., 2021) increased high-density lipoprotein cholesterol (HDL-C) levels and decreased the levels of triglyceride (TG), total cholesterol (TC), and low-density lipoprotein cholesterol (LDL-C).

#### 5.2.2 TCM injections for CMVD treatment

Many clinical studies have shown that TCM injections can play an important role in improving clinical symptoms, enhancing coronary microcirculation function, and regulating abnormal biomarkers and other aspects in CMVD patients.

1) Danhong injection (Hu et al., 2014), Shuxuening injection (Wu et al., 2010), Shuxuetong injection (Mao et al., 2021), Yiqi Fumai Lyophilized injection (Guo et al., 2020), *Salvia miltiorrhiza* and ligustrazine hydrochloride injection (Yang and Zhang, 2013), Ginkgo Damo injection (Wang et al., 2010), ligustrazine injection (Yue et al., 2018), safflower yellow pigment (SYPI) injection (Wang and Wang, 2013), and sodium tanshinone ⅡA sulfonate injection (Zu et al., 2012) improved angina symptoms and/or ECGs. 2) Ginkgo Damo injection (Wang et al., 2010) improved TIMI blood flow grading. 3) Danhong injection (Hu et al., 2014) and compound *Salvia miltiorrhiza* injection (Liu and Gu, 2021) increased NO levels and decreased ET-1 levels; ligustrazine injection (Yue et al., 2018) and compound *Salvia miltiorrhiza* injection (Liu and Gu, 2021) improved FMD; Shuxuetong injection (Mao et al., 2021), ligustrazine injection (Yue et al., 2018), and compound *Salvia miltiorrhiza* injection (Liu and Gu, 2021) decreased the levels of hs-CRP, TNF-α, and IL-6; Danhong injection (Chen et al., 2018), Safflower Yellow Pigment injection (Wang and Wang, 2013), and sodium tanshinone IIA sulfonate injection (Zu et al., 2012) decreased hs-CRP levels; and Danhong injection (Hu et al., 2014; Chen et al., 2018) increased E-selectin levels and decreased thrombomodulin levels.

#### 5.2.3 TCM decoctions for CMVD treatment

Many clinical studies have shown that TCM decoctions can play an important role in improving clinical symptoms, improving exercise tolerance and quality of life, enhancing coronary microcirculation function, and regulating abnormal biomarkers and other aspects in CMVD patients.

1) Liqi Huatan Huoxue formula (Wang et al., 2009), Huayu Fuyuan capsule (Peng and Yao, 2019), Huoxue Tongmai Yixin decoction (Li et al., 2020), Huoxue Zhuyu decoction (He et al., 2020), Fufang Wenban decoction (Jiang, 2021), Shenwu Guanxin granule (Sun et al., 2007), Bushen Huoxue granule (Wen et al., 2016), Tongmai Zhuyu decoction (Su and Luo, 2014), Yiqi Huoxue (Tian et al., 2015), Yiqi Huoxue Huatan formula granule (Zhou, 2018), Qingling decoction (Lin et al., 2011), Yishen Jieyu Tongmai decoction (Chen and Song, 2019), Qihong powder (Jiang et al., 2021), Yixin yin (Long et al., 2010), Yiqi Tongluo recipe (Wu et al., 2016), Yiqi Huoxue Huatan recipe (Huang et al., 2019), Yiqi Huoxue recipe (Feng et al., 2013), Wenyang Huoxue decoction (Xue et al., 2014), Tongluo Ningxin recipe (Li, 2018), Danshen decoction (Chen et al., 2021), Danggui Sini decoction (Cui et al., 2021), Erxian decoction (Zhang et al., 2012), Zhishi Xiebai Guizhi decoction (Shi and Cui, 2019), and Xuefu Zhuyu decoction (Zhu, 2019) improved angina symptoms and/or ECG results; Wenyang Huoxue decoction (Xue et al., 2014) reduced the nitroglycerin dosage needed; Huoxue Tongmai Yixin decoction (Li et al., 2020), Yixin yin (Long et al., 2010), Danggui Sini decoction (Cui et al., 2021), and Erxian decoction (Zhang et al., 2012) improved exercise tolerance; Qihong powder (Jiang et al., 2021), Yixin yin (Long et al., 2010), and Yiqi Tongluo recipe (Wu et al., 2016) improved quality of life. 2) Huoxue Tongmai Yixin decoction (Li et al., 2020) and Qihong powder (Jiang et al., 2021) improved CFRs; Zhima Xiaotan Huoxue recipe (Liu Q. et al., 2021), Yixin yin (Long et al., 2010), Yiqi Tongluo recipe (Wu et al., 2016), Yiqi Huoxue Huatan recipe (Huang et al., 2019), and Tongluo Ningxin recipe (Li, 2018) improved TIMI blood flow grading. 3) Liqi Huatan Huoxue formula (Wang et al., 2009), Huayu Fuyuan capsule (Peng and Yao, 2019), Huoxue Tongmai Yixin decoction (Li et al., 2020), Huoxue Zhuyu decoction (He et al., 2020), Fufang Wenban decoction (Jiang, 2021), Bushen Huoxue granule (Wen et al., 2016), Yiqi Huoxue (Tian et al., 2015), Qingling decoction (Lin et al., 2011), Qihong powder (Jiang et al., 2021), Yiqi Huoxue Huatan recipe (Huang et al., 2019), Wenyang Huoxue decoction (Xue et al., 2014), and Xuefu Zhuyu decoction (Zhu, 2019) increased NO levels and decreased ET-1 levels; Yiqi Tongluo recipe (Wu et al., 2016) improved FMD; Danqi Tongmai capsule (Ren et al., 2019) significantly decreased the levels of plasma ET-1, AngⅡ, and IL-6; Zhima Xiaotan Huoxue recipe (Liu Q. et al., 2021), Qihong powder (Jiang et al., 2021), Tongluo Ningxin recipe (Li, 2018), and Xuefu Zhuyu decoction (Jiang, 2021) significantly decreased the levels of IL-6 and hs-CRP; and Wenyang Huoxue decoction (Xue et al., 2014) and Xuefu Zhuyu decoction (Zhu, 2019) significantly decreased the TG, TC, and LDL-C levels and increased HDL-C levels.

In summary, clinical evidence indicates that TCM is beneficial for treating CMVD in 1) relieving the main clinical symptoms of angina pectoris, including decreasing the frequency of angina pectoris attacks, shortening the duration of angina pectoris, and alleviating the pain due to angina pectoris; improving the total effective rates of TCM syndromes; improving the quality of life of patients, including improving Seattle Angina Questionnaire scores and improving anxiety and depression; improving ECG results, including extending the total treadmill exercise times, enhancing the time of ST-segment depression of 1 mm, decreasing the maximum amplitude of ST-segment depression, and reducing the descending degree of the ST-T segment; and reducing the nitroglycerin dosage needed; 2) enhancing coronary microcirculation functions, including decreasing TIMI blood flow grading, increasing CFRs, and reducing IMRs; improving cardiac function, including decreasing cTnI and CK-MB levels and increasing EF levels; and 3) protecting vascular endothelial functions, including decreasing ET-1 levels, increasing NO levels, and improving FMDs; inhibiting inflammatory responses; decreasing the levels of hs-CRP, CRP, TNF-α, IL-1, IL-2, and IL-6; and improving blood lipids, which include decreasing the TG, TC, and LDL-C levels and increasing HDL-C levels.

## 6 Frequency of commonly used chinese herbs from TCM compounds

According to the statistics provided in Table 1, the most frequently used herb among the 107 Chinese herbs studied was Danshen (*Salvia miltiorrhiza* Bunge). The top 10 Chinese herbs that are ranked by their relatively high usage frequencies are shown in Table 3. Table 3 shows that the top ten Chinese herbs are mainly blood-activating and stasis-removing drugs.

**TABLE 3:** The top 10 Chinese herbs ranked with relatively high usage frequency.

No.	Chinese name	Latin name	Frequency
1	Danshen	*Salvia miltiorrhiza* Bunge	34
2	Huangqi	*Astragalus mongholicus* Bunge	21
3	Chuanxiong	*Conioselinum anthriscoides “Chuanxiong”*	21
4	Honghua	*Carthamus tinctorius* L*.*	15
5	Chishao	*Paeonia anomala* subsp*. veitchii* (Lynch) D.Y.Hong & K.Y.Pan	15
6	Danggui	*Angelica sinensis* (Oliv.) Diels	14
7	Sanqi	*Panax notoginseng* (Burkill) F.H.Chen	14
8	Shuizhi	*Hirudo*	14
9	Renshen	*Panax ginseng* C.A.Mey	13
10	Xiebai	*Allium chinense* G.Don	13

## 7 Potential action mechanisms of TCM for CMVD

### 7.1 Exploring the potential action mechanisms of TCM for CMVD based on clinical research

Coronary microcirculation disturbance (CMCD) is the main cause of myocardial ischemia in patients with CMVD (Padro et al., 2020). Vascular endothelial dysfunction is the main pathological mechanism of CMVD, and improving vascular endothelial dysfunction is one of the most important strategies in the treatment of CMVD (Ford et al., 2018; Kaski et al., 2018; Mangiacapra et al., 2020). CFRs, IMRs, and TIMI blood flow grading are commonly used methods to evaluate coronary microvascular function (Gibson et al., 2000; Gould, 2009; Cuculi et al., 2014). CFR is one of the sensitive indices that reflect changes in coronary hemodynamics, and it is also an index to evaluate the reserve functions of the coronary arteries and microcirculation. In CMVD without occlusive epicardial coronary artery disease, a decrease in CFR is a marker of CMCD (Gould, 2009). A decrease in CFR was associated with adverse cardiovascular events (Gibson et al., 2000; Cuculi et al., 2014). IMR can accurately reflect the pathological changes in microcirculation, and it is a specific method used for assessing CMCD (Murthy et al., 2014). IMR can specifically evaluate the microvascular functions of the distal ends of stenotic lesions and can accurately predict myocardial perfusion levels, ventricular remodeling, and cardiac function recovery after reperfusion therapy in AMI (Murthy et al., 2012; Choi et al., 2021). The normal value of IMR has not been universally determined, and IMRs >25 indicate the presence of CMD (Fearon et al., 2008). Clinical studies show that TCM can increase CFRs, decrease IMRs, and improve TIMI blood flow grading and correct TIMI frames in patients with CMVD, which suggests that the mechanism of TCM treatment of CMVD may be related to the improvement of CMCD.

The endothelium is key to maintaining intravascular homeostasis, and endothelial function impairment is the main etiological basis for the occurrence and development of arteriosclerosis. Therefore, determinations of vascular endothelial function are of great clinical significance in the prevention and treatment of cardiovascular diseases (Godo and Shimokawa, 2017). ET-1 and NO, a pair of antagonistic vasoactive substances synthesized by the endothelium, are commonly used markers of endothelial cell function. The dynamic equilibrium between them is important for maintaining vascular tension and stability of the cardiovascular system. The brachial artery FMD technique was first developed by Celermajer et al. (1992). It is a noninvasive, high-frequency ultrasound method used to evaluate vascular endothelial function. FMD can reflect the overall functional changes of the vascular endothelium, and early detection can reveal the early progression of arteriosclerosis. Clinical studies have shown that TCM can significantly increase NO and NO/ET levels, decrease ET-1 levels, and improve FMD and vascular endothelial function. It is suggested that the protection of vascular endothelial function may be one of the mechanisms of TCM treatment of CMVD.

Inflammatory reactions play an important role in the development of CMD. Inflammatory factors not only cause endothelial injury and intimal thickening but also decrease NO and prostacyclin synthesis in endothelial cells and activate immune cells to release human ET and ET-like immune complexes, ultimately leading to endothelial dysfunction (Marroquin et al., 2005; Arroyo-Espliguero and Kaski, 2006). Clinical studies have shown that the levels of IL-6, hs-CRP, WBC, and neutrophil-lymphocyte in the peripheral blood of patients with CMVD are significantly higher than those of healthy subjects, which suggests that endothelial dysfunction induced by inflammatory reactions may be one of the pathogenic mechanisms of CMVD (Demirkol et al., 2014; Jia J. Z. et al., 2019). TCM can significantly reduce the levels of CRP, hs-CRP, IL-1, IL-6, TNF-α, and other inflammatory factors, suggesting that the inhibition of inflammatory reactions and protection of vascular endothelial function may be one of the mechanisms of TCM treatments of CMVD.

### 7.2 Exploring the potential action mechanisms of TCM for CMVD based on basic experimental research

Table 3 shows that the top ten Chinese herbs used to treat CMVD consist mainly of blood-activating, stasis-removing, and qi-reinforcing drugs. Next, we discuss the potential mechanisms of Chinese herbs such as Danshen (*Salvia miltiorrhiza* Bunge), Honghua (*Carthamus tinctorius* L.), Huangqi (*Astragalus mongholicus* Bunge), and Danghui (*Angelica sinensis* (Oliv.) Diels) in the treatment of CMVD.

#### 7.2.1 Danshen (*salvia miltiorrhiza* bunge)

*Salvia miltiorrhiza* Bunge is the most commonly used traditional Chinese medicine for the clinical treatment of CMVD. Modern pharmacological studies have shown that *Salvia miltiorrhiza* Bunge has anti-inflammatory, antioxidant, anti-atherosclerotic, anti-coagulant, and anti-thrombotic effects, which regulate blood lipids, increase coronary blood flow, improve microcirculation, and protect vascular endothelial function. Liu et al. (2008) reported that *Salvia miltiorrhiza* Bunge could inhibit platelet aggregation and activation, prevent microthrombosis, promote nitric oxide production, dilate blood vessels, increase blood flow, and improve blood hypercoagulability, thus playing an anticoagulant role. Tanshinone IIA, one of the most abundant components of tanshinone, can alleviate pathological injury of cardiac tissue, attenuate myocardial damage, reduce myocardial infarct sizes, and promote the recovery of cardiac function (Zhang et al., 2010). In addition, tanshinone IIA sodium sulfonate could increase coronary flow, improve myocardial hypoxia tolerance, improve myocardial metabolic disorders, and effectively inhibit platelet aggregation and antithrombosis in patients with coronary heart failure (Wang and Liu, 2013; Qu et al., 2016). Salvianolic acid is a water-soluble phenolic acid extracted from *Salvia miltiorrhiza* Bunge that significantly reduced cardiomyocyte injury, reduced myocardial inflammatory cell infiltration, and protected the myocardium in a rat model of myocardial ischemia–reperfusion injury (Qiu et al., 2017). Meng et al. (2014) found that salvianolic acid salt plus vitamin D3 injections could alleviate atherosclerosis induced by high-fat diets by inhibiting the inflammatory process. Its protective mechanism in atherosclerosis is closely related to the inhibition of oxidative stress and inflammatory responses and the improvement of endothelial dysfunction (Song et al., 2019). In addition, it was found (Wang et al., 2018) that the polysaccharides of *Salvia miltiorrhiza* Bunge also exhibited good antioxidant activities. Compared with vitamin C, Salvia miltiorrhiza polysaccharides could scavenge more than 90% of free radicals and could be used to prevent cell damage caused by free radicals and intracellular reactive oxygen species (ROS). Cong and Yu (2015) reported that Danshensu could reduce MDA contents, increase SOD activities, enhance cellular oxygen radical scavenging abilities, and improve vascular endothelial cell viabilities in vascular endothelial cells with H2O2-induced injuries and play a protective role against H2O2-induced oxidative damage in vascular endothelial cells. Salvianolic acid B, as one of the main water-soluble components of *Salvia miltiorrhiza* Bunge, has significant anti-inflammatory and cardiovascular protective effects. Hu et al. (2020) established a myocardial ischemia model and H9C2 cell inflammation model in rats and treated them with different concentrations of salvianolic acid B. Results showed that salvianolic acid B could significantly decrease acute myocardial ischemic injury in rats, significantly inhibit the production of ROS in H9C2 cells, increase the mitochondrial membrane potential, inhibit activation of the NLRP3 inflammasome, and inhibit apoptosis. Animal experiments revealed that salvianolic acid A could exert antiapoptotic effects during myocardial ischemia–reperfusion by activating extracellular signal-regulated kinase 1/2 (ERK1/2) and inhibiting Jun kinase (JNK) (Xu et al., 2014). In addition, *Salvia miltiorrhiza* Bunge also has hypolipidemic effects. Lim et al. (2017) established a hyperlipidemic mouse model with a high-fat diet and investigated the lipid-regulating mechanisms of methanol extracts of *Salvia miltiorrhiza* Bunge. It was found that the methanol extracts of *Salvia miltiorrhiza* Bunge ameliorated hyperlipidemia in mice fed a high-fat diet mainly by inhibiting the increase in serum triacylglycerol levels.

#### 7.2.2 Honghua (*carthamus tinctorius* L.)

*Salvia miltiorrhiza* Bunge and *Carthamus tinctorius* L. were used as a herb pair to treat cardiovascular diseases. Bai et al. (2021) reported that they could reduce the sizes of myocardial infarcts, alleviate ischemic injuries, and inhibit cardiomyocyte apoptosis in a myocardial ischemia/reperfusion injury (MIRI) model *in vitro*. Danhong injections are composed of *Salvia miltiorrhiza* Bunge and *Carthamus tinctorius* L. Modern research has found that Danhong injections can protect against cardiomyocyte injury, inhibit cardiomyocyte apoptosis, and improve the cell survival rates of myocardial cells (Duan et al., 2015). In addition, Yuan (2019) reported that Danhong injections protected against myocardial ischemia–reperfusion injuries in rabbits *via* antioxidation and the inhibition of cardiomyocyte apoptosis. Hydroxysafflor yellow A, one of the main active ingredients of *Carthamus tinctorius* L*.*, alleviates MI/RI damage to both heart structures and functions (Ye et al., 2021). In addition, safranal, an active ingredient extracted from saffron, exerts a protective effect on the cardiovascular system. Wang H. et al. (2021) reported that safranal could increase the viability of H9c2 cardiac myoblasts and alleviate H/R-induced H9c2 cardiac myoblast injuries *via* the PI3K/AKT/GSK3β signaling pathway.

#### 7.2.3 Huangqi (*astragalus mongholicus* bunge) and danghui (*angelica sinensis* (Oliv.) diels)

*Astragalus mongholicus* Bunge and *Angelica sinensis* (Oliv.) Diels are commonly used as couplet medicine in the clinical treatment of CMVD. Modern pharmacological studies have shown that *Astragalus mongholicus* Bunge–*Angelica sinensis* (Oliv.) Diels improved blood circulation, had anti-inflammation and antioxidation effects, and protected the vascular endothelium. By promoting the expression of endothelial nitric oxide synthase (eNOS) and phosphorylation of protein kinase B (PKB/Akt), *Astragalus mongholicus* Bunge–*Angelica sinensis* (Oliv.) Diels can promote nitric oxide (NO) release and diastolic blood vessels, which protect the endothelium. It can also inhibit the apoptosis of vascular endothelial cells by inhibiting inducible NOS (iNOS) expressions, improving the expressions of local inflammatory response factors in blood vessels, and inhibiting intimal hyperplasia due to endothelial injury (Xiang et al., 2022). Astragaloside IV is one of the major active components of *Astragalus membranaceus*, which can reduce cardiomyocyte injuries and attenuate cardiomyocyte apoptosis induced by hypoxia/reoxygenation (H/R) *via* the inhibition of the calcium-sensing receptor (CaSR)/extracellular signal-regulated kinase 1/2 (ERK1/2), and related apoptotic signaling pathways (Yin et al., 2019). Astragalus polysaccharide can significantly increase human cardiac microvascular endothelial cell viabilities and reduce apoptosis levels due to H/R injury (Xie et al., 2016).

#### 7.2.4 Other chinese herbs

*Conioselinum anthriscoides “Chuanxiong*,” *Paeonia lactiflora Pall*, *Panax notoginseng* (Burkill) F.H. Chen, and *Hirudo* are all blood-activating and stasis-resolving medicines. Modern pharmacological studies have shown that they all exhibit anti-myocardial ischemia–reperfusion injury, blood flow alteration, anti-atherosclerosis, platelet aggregation inhibition effects, and scavenge oxygen free radicals and also have anti-inflammation, vasodilation, protection of vascular endothelium, and promotion of angiogenesis effects (Liu Y. C. et al., 2021; Wu et al., 2021; Zhang and Ma, 2021; Jiang et al., 2022). Tetramethylpyrazine is one of the main active components of *Conioselinum anthriscoides “Chuanxiong.”* Tetramethylpyrazine can significantly reduce myocardial infarction sizes, improve myocardial function, reduce cardiomyocyte apoptosis, and provide significant protective effects on cardiomyocytes after ischemia–reperfusion. Its mechanism may be through the regulation of Janus kinase signal transducers 2/signal transducers, activators of the transcription 3 signaling pathway, and mitochondrial autophagy (Cheng et al., 2021). Total paeony glycoside improved the survival rate of cardiomyocytes, inhibited cardiomyocyte apoptosis, and protected H9c2 cardiomyocytes from H/R injury (Shen et al., 2018). Ginsenoside Rb1, ginsenoside Rg1, ginsenoside Re, and notoginsenoside R1 are the major, active components of *Panax notoginseng* (Burkill) F.H. Chen. Studies have reported (Cui et al., 2017; Li et al., 2018) that ginsenoside Rb1 and ginsenoside Rg1 can decrease I/R-induced myocardial infarct sizes, ameliorate cardiomyocyte injuries, restore myocardial blood flow, and improve heart function. Ginsenoside Re and notoginsenoside R1 can increase NO release in vascular endothelial cells and stimulate vasodilation (Huang et al., 2005; Wang et al., 2016). As a representative TCM for breaking blood and expelling blood stasis, *Hirudo* has a significant antithrombotic effect. Li et al. (2021) found that *Hirudo* extracts could inhibit venous thrombosis through antioxidant effects, thus achieving antithrombotic effects. Atherosclerosis is the pathological basis of many cardiovascular diseases. *Hirudo* and its extracts can exert anti-atherosclerotic effects by regulating lipid metabolism, protecting endothelial cells, and inhibiting smooth muscle proliferation. Chen et al. (2013) found that hirudin could exert an anti-atherosclerotic effect by decreasing the levels of inflammatory factor TNF-α and inhibiting the proliferation of smooth vascular muscle cells.

In conclusion, TCM may achieve the purpose of CMVD treatment through anti-inflammatory and antioxidant effects, improve vascular endothelial function, protect cardiomyocytes, and improve coronary microcirculation.

## 8 Scientific problems and research directions of CMVD

Although significant progress has been made in the basic and clinical research of CMVD, many scientific issues in this field remain to be solved. 1) Nonuniform clinical classifications: in the early literature, patients exhibiting chest pain without significant coronary angiography abnormalities were classified as having syndrome X. With the improvements in clinical research and diagnostic techniques, the clinical classification of CMVD has become increasingly detailed. Reasonable clinical classifications are helpful to elucidate the pathogenesis, establish diagnostic criteria and treatment choices, and determine prognoses. Establishing a more reasonable clinical classification of CMVD needs further study. 2) Unknown pathogeneses: because the clinical phenotype of CMVD is very complex, many CMVD mechanisms have been proposed, including endothelial cell-dependent and non-dependent vasodilation abnormalities, microvascular spasms, microvascular embolization, extravascular compression, and other mechanisms (Ford et al., 2018). What are the pathogenic differences among the clinical phenotypes of CMVD? What are the key factors that influence these mechanisms? Do interventions involving these mechanisms improve clinical symptoms and patient prognoses? These questions remain unanswered. In order to explore the mechanisms of CMVD, it is necessary to establish an animal model similar to pathological CMVD changes in humans. However, there is still a lack of animal models that can simulate human CMVD. 3) Inadequate clinical diagnoses: because the diameters of coronary microvessels are smaller than the resolution of existing imaging techniques, the morphology of these vessels cannot be observed by clinicians. Therefore, laboratory diagnoses of CMVD are mainly based on measurements of coronary microvascular function. Although the value of invasive examinations in the diagnosis of coronary artery function is well known, there are a few clinical applications for various reasons, which lead to a lack of CMVD diagnoses. (4) Unclear drug efficacies: currently, although there are small randomized clinical studies or nonrandomized observational studies in the literature that focus on CMVD and end-point coronary microvascular function, there is still a lack of results from large randomized clinical trials with CMVD as the subject and cardiovascular events as the endpoint of the observations (Crea et al., 2014). CMVD treatments are still limited to controlling the traditional risk factors for arteriosclerosis, improving lifestyles, and relieving angina symptoms. Therefore, the treatments that can decrease the incidence of cardiovascular events in CMVD have not been determined.

In view of the above scientific challenges, we suggest that future research should focus on the following areas: 1) studying animal models that can simulate human CMVD; 2) exploring the key molecules and targets of CMVD development and studying CMVD pathogenesis by using genomics, transcriptomics, and proteomics techniques to search for molecular markers and intervention targets; 3) developing highly sensitive and specific serological markers that can predict and detect CMVD and developing molecular imaging techniques that can reveal the extent of CMVD; and 4) conducting large-sample, multicenter, randomized clinical trials with cardiovascular event endpoints to validate the potential for new CMVD-specific drugs and therapies to improve clinical endpoints and establish evidence-based medical treatments. Objective and accurate evaluations of CMVD are of great value for both CMVD diagnosis and searching for new intervention measures. In the future, more in-depth research on the diagnosis, treatment, and pathological mechanism of CMVD is expected to be conducted to establish a better and more accurate diagnosis and treatment strategies.

## 9 Conclusion and perspectives

Cardiovascular disease is one of the main causes of human death. Previous studies have shown that myocardial ischemia is mainly caused by epicardial coronary artery occlusion. However, in some patients with angina pectoris, coronary angiography (CAG) did not reveal occlusive disease, which suggested that myocardial ischemia may be the cause of coronary microcirculation dysfunction (Padro et al., 2020). Although the CAG technique has resulted in great progress in recent decades and the diagnostic accuracy of subepicardial coronary artery stenosis has been improved, the diagnostic sensitivity of CAG for myocardial ischemia is still limited. Studies have shown that half of the patients with known or suspected angina who receive CAG have nonobstructive coronary stenosis, which may be related to CMVD (Ford et al., 2020). CMVD is associated with an increased risk of MACEs (Jespersen et al., 2012; Maddox et al., 2014). The coronary angiography results were associated with significantly higher rates of cardiovascular events and all-cause mortality in patients with angina and with nonobstructive coronary artery disease than in the controls. The main cause of the poor prognoses in these angina patients may be related to CMVD, as researchers have determined (Taqueti et al., 2017; Taqueti and Di Carli, 2018). Therefore, the detection and treatment of CMVD have important clinical significance. There are no consensus on CMVD treatments, clinical practices for patients to improve symptoms, and intervention strategies for cardiovascular risk factors. Although CMVD has received increasing attention in the cardiovascular field, the etiological screening of myocardial ischemia is still limited to assessments of the epicardial coronary arteries, and assessments of coronary microcirculation have not received enough attention. At present, there is no large-scale clinical study of CMVD with cardiovascular outcomes. In summary, CMVD is an important problem in the cardiovascular field. Although the study of CMVD has been deepened in modern medicine, the diagnosis, evaluation, and treatment of CMVD still face great challenges.

In recent years, the understanding of CMVD with respect to traditional Chinese medicine has been greatly improved, and many clinical studies have been conducted. Treatments based on syndrome differentiation using CPMs, TCM injections, and TCM decoctions can effectively relieve the clinical symptoms of patients, improve their quality of life and exercise tolerance, improve their long-term prognoses, and decrease their readmission rates, which provide new ideas and directions for the study of CMVD. However, due to the flexibility of treatment based on syndrome differentiation, the standards for syndrome differentiation and reasonable diagnosis and treatment schemes have not been unified, making it difficult to introduce TCM in clinics. At present, most clinical studies of traditional Chinese medicine for CMVD are characterized by inconstant designs, small sample sizes, short observation times, and a lack of large-scale multicenter prospective randomized control studies; therefore, the results of such studies cannot be extended to a wide range of clinical applications.

In addition, TCM compounds have multicomponent, multitarget, and multi-pathway synergistic actions. At present, research on CMVD mainly focuses on the action of one extract or one part of a single drug, which lays a foundation for research on the material basis of TCM compound prescriptions. Studying single ingredients ignores the relationships among drug taste, drug properties, and symptoms, and studies of compound prescriptions have mostly remained at the pharmacodynamic stage. Pharmacodynamic evaluations mostly use the evaluation system of a single target of chemical drugs for reference and neglect the characteristics of synergistic effects and multipoint fine-tuning. However, it should be noted that compound TCM prescriptions are the main form of clinical TCM use, and the clinical applications of TCM should be based on syndrome differentiation. At present, little attention has been paid to the compatibility of effective components, interactions among components, and the differences among different components. As a result, the current approach fails to emphasize the concept of compatibility of TCM to increase efficacy and decrease toxicity.

Therefore, future studies of TCM treatment of CMVD should begin from the following three perspectives. 1) For the theoretical research aspect, it is necessary to systematize and summarize the relevant ancient books and existing research to further improve the understanding of CMVD in TCM. 2) With regard to clinical research, we need to strengthen scientific and normative research and conduct large-sample, multicenter randomized clinical trials with the endpoints of cardiovascular events, evaluate the potential for CMVD-specific new drugs and therapies to improve clinical endpoints, and conduct efficacy and safety evaluations to provide stronger evidence-based medical evidence. 3) In basic research, we should use network pharmacology, metabolomics, molecular biology, proteomics, and other new research methods to explain the mechanisms of TCM in treating CMVD at the metabolite, gene, and protein levels to provide a new strategy for controlling CMVD.

In summary, CMVD still presents a thorny clinical problem. TCM has great therapeutic potential with respect to CMVD, but more large-scale and in-depth clinical and animal studies are needed to promote the clinical applications of TCM in CMVD.
